# Hepatocyte Smoothened Activity Controls Susceptibility to Insulin Resistance and Nonalcoholic Fatty Liver Disease

**DOI:** 10.1016/j.jcmgh.2022.12.008

**Published:** 2022-12-16

**Authors:** Tianyi Chen, George Dalton, Seh-Hoon Oh, Raquel Maeso-Diaz, Kuo Du, Rachel A. Meyers, Cynthia Guy, Manal F. Abdelmalek, Ricardo Henao, Paolo Guarnieri, Steven S. Pullen, Simon Gregory, Joseph Locker, J. Mark Brown, Anna Mae Diehl

**Affiliations:** 1 Department of Medicine, Duke University, Durham, North Carolina; 2 Boehringer Ingelheim Pharmaceuticals Inc, Ridgefield, Connecticut; 3 Department of Pathology, University of Pittsburgh, Pittsburgh, Pennsylvania; 4 Department of Cardiovascular and Metabolic Sciences, Cleveland Clinic Lerner Research Institute, Cleveland, Ohio

**Keywords:** hedgehog, nonalcoholic fatty liver disease, metabolic syndrome, AMPK, AMP kinase, AST, aspartate aminotransferase, ATP, adenosine triphosphate, BMI, body mass index, DUHS, Duke University Health System, GSEA, gene set enrichment analysis, GO, Gene Ontology, IR, insulin receptor, IRS, insulin receptor substrate, KO, knockout, MetS, metabolic syndrome, mRNA, messenger RNA, mTOR, mammalian target of rapamycin, NAFLD, nonalcoholic fatty liver disease, NASH, nonalcoholic steatohepatitis, NPC, nonparenchymal cell, Ptch, Patched, RNA-seq, RNA sequencing, Smo, Smoothened, SREBP1c, sterol regulatory element binding protein-1c

## Abstract

**Background & Aims:**

Nonalcoholic steatohepatitis (NASH), a leading cause of cirrhosis, strongly associates with the metabolic syndrome, an insulin-resistant proinflammatory state that disrupts energy balance and promotes progressive liver degeneration. We aimed to define the role of Smoothened (*Smo*), an obligatory component of the Hedgehog signaling pathway, in controlling hepatocyte metabolic homeostasis and, thereby, susceptibility to NASH.

**Methods:**

We conditionally deleted *Smo* in hepatocytes of healthy chow-fed mice and performed metabolic phenotyping, coupled with single-cell RNA sequencing (RNA-seq), to characterize the role of hepatocyte *Smo* in regulating basal hepatic and systemic metabolic homeostasis. Liver RNA-seq datasets from 2 large human cohorts were also analyzed to define the relationship between *Smo* and NASH susceptibility in people.

**Results:**

Hepatocyte *Smo* deletion inhibited the Hedgehog pathway and promoted fatty liver, hyperinsulinemia, and insulin resistance. We identified a plausible mechanism whereby inactivation of *Smo* stimulated the mTORC1-SREBP1c signaling axis, which promoted lipogenesis while inhibiting the hepatic insulin cascade. Transcriptomics of bulk and single *Smo-*deficient hepatocytes supported suppression of insulin signaling and also revealed molecular abnormalities associated with oxidative stress and mitochondrial dysfunction. Analysis of human bulk RNA-seq data revealed that *Smo* expression was (1) highest in healthy livers, (2) lower in livers with NASH than in those with simple steatosis, (3) negatively correlated with markers of insulin resistance and liver injury, and (4) declined progressively as fibrosis severity worsened.

**Conclusions:**

The Hedgehog pathway controls insulin sensitivity and energy homeostasis in adult livers. Loss of hepatocyte Hedgehog activity induces hepatic and systemic metabolic stress and enhances susceptibility to NASH by promoting hepatic lipoxicity and insulin resistance.

## Graphical abstract

**Figure:**
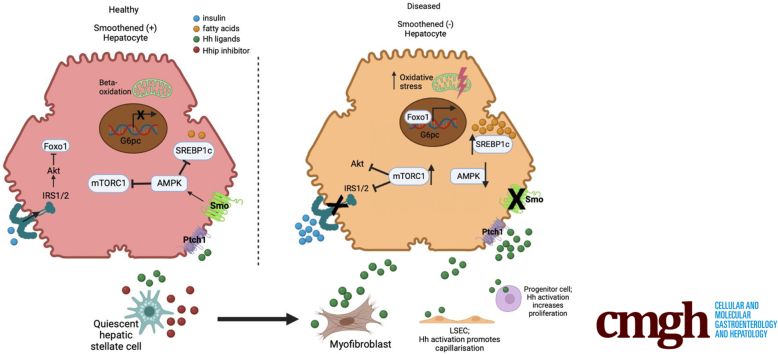

**Summary**

Hepatocytes require Smoothened, a fat-regulated signaling protein, to protect themselves from metabolic stress, and mice rapidly develop fatty liver disease and prediabetes when Smoothened is inhibited. Analysis of human livers suggests Smoothened also regulates susceptibility to these conditions in people.

Nonalcoholic fatty liver disease (NAFLD), the liver correlate of metabolic syndrome (MetS), occurs in 25% of adults worldwide and is now the leading cause of cirrhosis, liver failure, and liver cancer in many countries.^1^ The risk for premature mortality from all causes is also significantly increased in NAFLD patients with advanced liver fibrosis.^2^ These poor outcomes rarely ensue unless hepatocyte lipid accumulation is accompanied by recurrent bouts of increased hepatocyte death and related wound healing responses (together dubbed nonalcoholic steatohepatitis [NASH]). The pathogenic mechanisms for NASH are poorly understood, but there is little argument that it associates with metabolic stress.^3^

Protection from metabolic stress is a critical function of the liver. Healthy livers respond to fluctuations in energy supply by modulating energy production to match the body’s demand for energy. Hepatocytes play a central role in maintaining systemic energy homeostasis. These cells are spatially organized along perfusion gradients to optimize progressive extraction of blood-borne toxins and nutrients, while sequentially enriching blood that is coursing through the liver with energy substrates and other factors that modulate the metabolic activities of both resident liver cells and extrahepatic tissues.^4^ The mechanisms that permit functional heterogeneity within the hepatocyte population are not well understood. Growing evidence indicates that these processes are quite malleable and involve dynamic zonal variations in morphogenic signaling pathways that also regulate state transitions and viability of adult hepatocytes, such as Wnt and Notch.^5^

In many cell types, both Wnt and Notch signaling interface with Hedgehog, a morphogenic signaling pathway that is exquisitely sensitive to lipids.^6^^,^^7^ Briefly, all 3 Hedgehog ligands are palmitoylated and modified by cholesterol. Patched (*Ptch*), the cell surface receptor for these ligands, senses cholesterol and regulates the activity of the pathway’s signaling-competent G protein-coupled co-receptor, Smoothened (*Smo*), by gating *Smo*’s direct interaction with cholesterol. *Smo* activity is also directly modulated by endogenous oxysterols and endocannabinoids that compete with cholesterol for *Smo* binding. The Hedgehog pathway is a proven regulator of state transitions in many of the cells that participate in hepatic wound healing responses, including hepatic stellate cells, immune cells, sinusoidal endothelial cells and bile duct cells (cholangiocytes).^8^, ^9^, ^10^, ^11^ Each of these cell types not only responds to Hedgehog ligands, but also can produce them; thus, Hedgehog-sensitive autocrine and paracrine interactions are central to liver repair.^12^, ^13^, ^14^

Hedgehog signaling is complex and includes a *Smo*-controlled canonical pathway that culminates in nuclear accumulation of the 3 activated *Gli* family transcription factors that control the expression of Hedgehog target genes.^15^ *Smo* also regulates noncanonical Hedgehog signaling and this latter function controls phosphorylation of AMP kinase (AMPK), a central regulator of cellular adenosine triphosphate (ATP) balance and, thus, energy homeostasis.^16^ Until recently, the Hedgehog pathway was thought to be silenced in healthy hepatocytes after birth, becoming reactivated only by oncogenic mutations or epigenetic events that occur relatively commonly in primary hepatocellular carcinomas. However, growing evidence suggests that Hedgehog, like Wnt and Notch, may remain active in subpopulations of healthy hepatocytes in order to help regulate hepatic metabolism. Matz-Soja et al^17^ have reported that a *Smo*-dependent *Gli* code constrains hepatocyte lipogenesis; subsequently, we demonstrated that hepatocyte *Smo* activity controls hepatic cholesterol metabolism and systemic homeostasis of cholesterol derivatives, such as bile acids.^18^ Findings from the 2 groups are summarized in Table 1. The recently documented effects of *Smo* on fatty acid and cholesterol metabolism suggest that dysregulated Hedgehog signaling in hepatocytes may be important in the pathogenesis of NAFLD and the MetS because both disorders strongly associate with dysfunctional lipid metabolism and defective responses to insulin and other lipid-regulating hormones.^19^ To evaluate this hypothesis, we conditionally deleted *Smo* specifically in hepatocytes of healthy chow-fed mice and performed metabolic phenotyping, coupled with single-cell analytical approaches to characterize the role of hepatocyte *Smo* activity in regulating hepatic and systemic metabolic homeostasis. We also analyzed liver RNA-sequencing (RNA-seq) datasets from 2 large human NAFLD cohorts to define the relationship between *Smo* activity and NAFLD susceptibility/severity in people. Our results reveal the importance of reduced *Smo* activity in the pathogenesis of NAFLD and the MetS.

**Table 1:** Summary of Findings From the Present Study and Gebhardt’s Group

	Hepatocyte-Specific Knockout	Fatty Liver/Steatosis	Insulin Resistance	Liver Injury/Inflammatory Signaling	Mitochondrial Dysfunction	Telomere Attrition	DNA Damage	Single-Cell Analysis	Human RNA-Seq Analysis
Matz-Soja et al^17^	—	Yes	—	—	Yes	—	—	—	—
Dalton et al^18^	Yes	Yes	—	—	—	—	—	—	—
Maeso-Diaz et al^20^	Yes	—	—	—	Yes	Yes	—	—	—
Present study	Yes	Yes	Yes	Yes	Yes	—	Yes	Yes	Yes

## Results

### Hepatocyte-Specific *Smo* Knockout Induces NAFLD and Insulin Resistance in Mice

**Figure 1:**
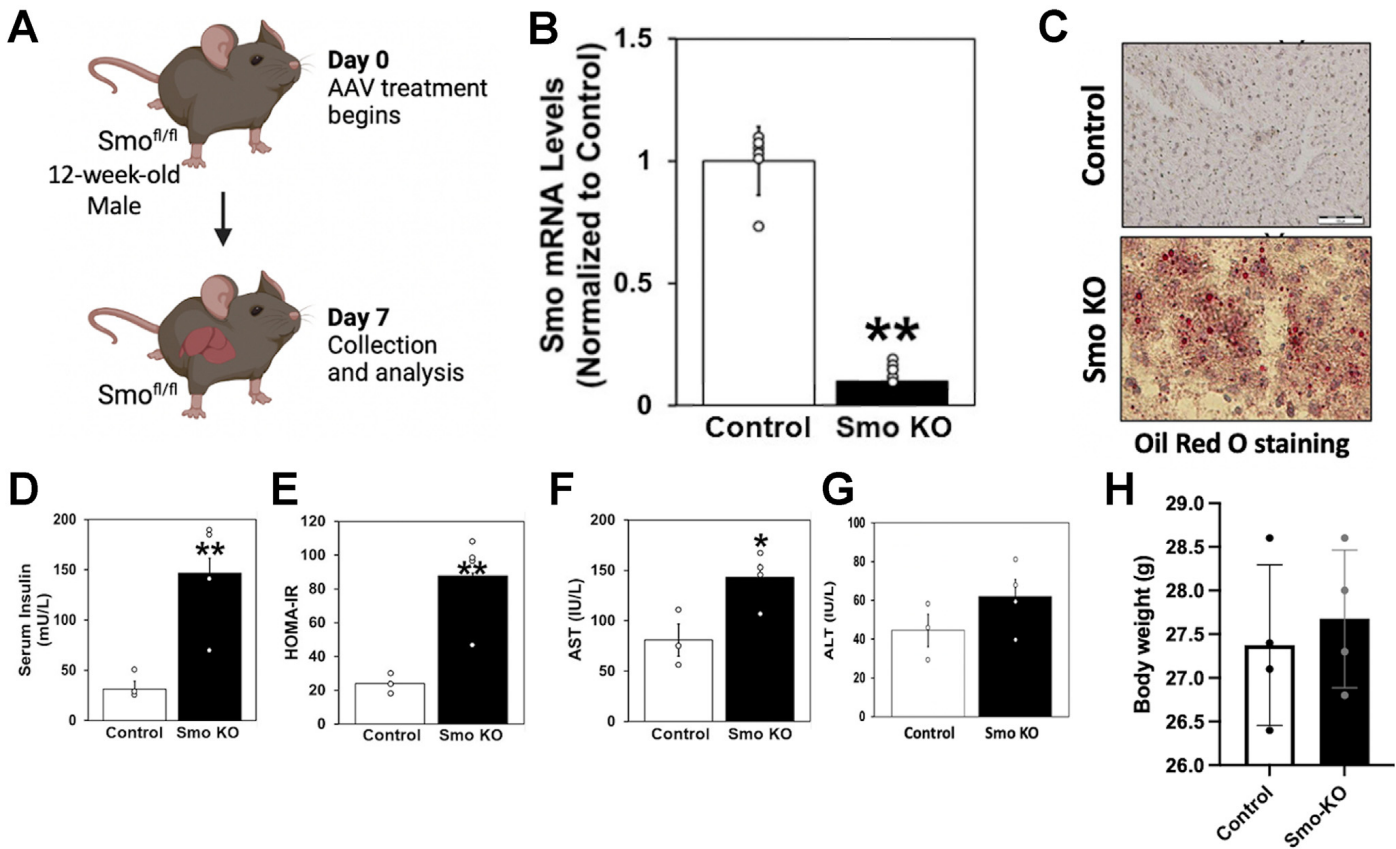
**Hepatocyte-specific *Smo*-KO induces NAFLD and insulin resistance in mice.** (*A*) Schematic of the *Smo*-KO study. (*B*) Quantitative reverse-transcription polymerase chain reaction (qRT-PCR) analysis of *Smo* in hepatocytes and liver nonparenchymal cells after AAV-TBG-CRE treatment of *Smo*^*flox/flox*^ mice. (*C*) Representative oil red staining in the mouse liver. Scale bars = 100 μm. (*D–G*) Quantification of serum insulin, Homeostatic Model Assessment for Insulin Resistance, AST, and alanine aminotransferase (ALT) after *Smo* knockout. (*H*) Body weights (g) were quantified. Data are displayed as mean ± SEM. Significance was determined using Student’s *t* test. The error bar indicates SEM. ∗*P* ≤ .05; ∗∗ P≤.01. AAV, adeno-associated virus; ns, not significant.

To define the role of *Smo* in healthy mature hepatocytes, we performed a conditional loss-of-function study by treating adult *Smo*^*flox/flox*^ mice with either AAV-TBG-Cre (*Smo* knockout [KO]) or AAV-TBG-Luciferase (vehicle control) (Figure 1*A*). This viral-based approach targeted hepatocytes specifically and demonstrated high efficiency as evidenced by >90% depletion of *Smo* messenger RNAs (mRNAs) in hepatocytes of *Smo*-KO mice compared with that of vehicle-treated control mice (Figure 1*B*). Consistent with our recent report that *Smo* deletion increased hepatic triglyceride and cholesterol content^18^ livers of mice with *Smo*-deficient hepatocytes stained strongly for Oil Red O, a marker of fatty livers (Figure 1*C*). The *Smo*-KO mice also exhibited hyperinsulinemia and an increased HOMR-IR index, suggesting they had developed insulin resistance and hepatic steatosis in parallel (Figure 1*D* and *E*). Additionally, serum levels of aspartate aminotransferase (AST) and alanine aminotransferase (Figure 1*F* and *G*) were elevated in *Smo*-KO mice, indicating that *Smo* deletion evoked lipotoxicity, a process that promotes NASH. We were intrigued by these phenotypes because they emerged in previously healthy nonobese mice within a week of *Smo* deletion. Mice were maintained on regular chow both before and after the adeno-associated virus administration and body weights after treatment were similar in *Smo*-KO mice and vehicle-treated control mice (Figure 1*H*), demonstrating that an obesogenic challenge was not necessary to provoke the observed phenotypes. Because the genetic alteration was elicited selectively in hepatocytes, the hyperinsulinemia noted in the *Smo*-KO mice may have been triggered by impaired insulin action in hepatocytes.

### *Smo* Impairs Hepatic Insulin Signaling and Promotes Lipogenesis by Activating the Mammalian Target of Rapamycin

**Figure 2:**
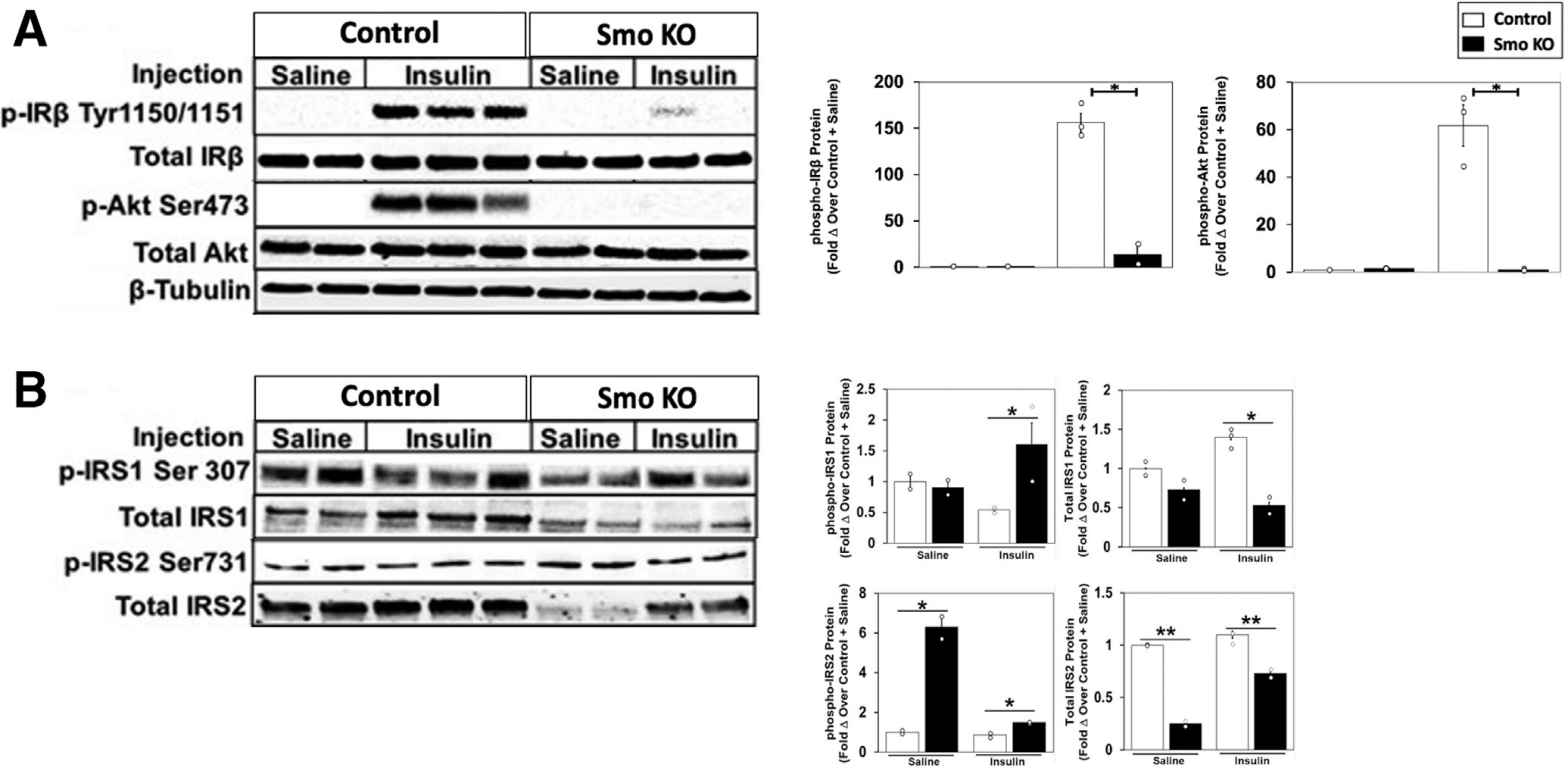
***Smo* deletion impairs hepatic insulin-IR signaling.** (*A*) Protein immunoblots of IRβ, Akt in control and *Smo*-KO mice treated with saline or insulin (left). Quantification of the intensity from the blots (right). (*B*) Protein immunoblots of pIRS1/2 and their total proteins in control and *Smo*-KO mice treated with saline or insulin (left). Quantification of the intensity from the blots (right). The error bar indicates SEM. ∗*P* ≤ .05; ∗∗ P≤.01.

**Figure 3:**
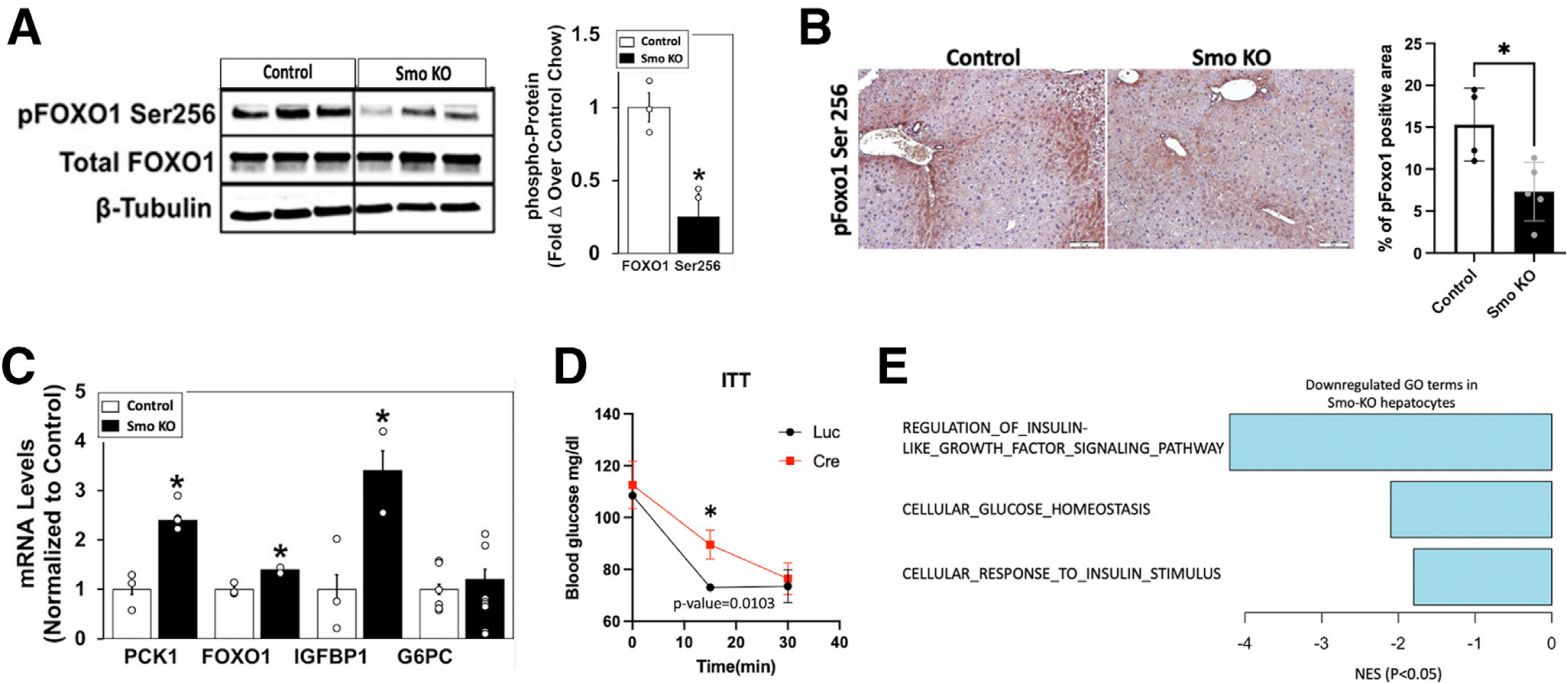
***Smo* deletion induces genes that promote hepatic gluconeogenesis and inhibits insulin-mediated suppression of blood glucose levels.** (*A*) Protein immunoblots of pFOXO1 and total FOXO1 in control and *Smo*-KO mice. (*B*) Representative staining of cytosolic pFOXO1-S256. Scale bars = 10 μm. (*C*) Quantitative reverse-transcription polymerase chain reaction (qRT-PCR) analysis of *Foxo1* and target genes. (*D*) Blood glucose levels (mg/dL) in control and *Smo*-KO mice after insulin treatment to assess insulin tolerance. (*E*) Enrichment analysis of bulk RNA-seq data showing downregulated GO terms in *Smo*-KO hepatocytes vs control hepatocytes. The error bar indicates SEM. ∗*P* ≤ .05; ∗∗ P≤.01.

To determine whether *Smo* inactivation is sufficient to suppress hepatic insulin signaling, we subjected another cohort of control and *Smo*-KO mice to an acute injection of insulin and then evaluated the phosphorylation state of key components in the insulin signaling cascade by performing immunoblots on collected liver tissues. *Smo* deletion abrogated insulin-stimulated phosphorylation of both the insulin receptor beta subunit (IRβ) and its downstream effector Akt (Figure 2*A*).^21^ IR substrates 1 and 2 (IRS1/2) are required to transduce signaling between IRs and Akt. Increased pIRS1-S307 and pIRS2-S731 have been previously linked to inhibition of this signal transduction.^22^^,^^23^ We showed that the IRS1 response was suppressed in insulin-treated *Smo*-KO livers reflected by marked reduction of IRS1 protein level as well as increased pIRS1-S307 (Figure 2*B*). Similarly, protein levels of IRS2 were significantly diminished in both saline- and insulin-treated *Smo*-KO livers, whereas pIRS2-S731 levels were markedly elevated (Figure 2*B*). Insulin functions to inhibit hepatic gluconeogenesis through phosphorylation and nuclear exclusion of Foxo1.^24^ *Smo*-KO livers had significantly less Foxo1 phosphorylation (Figure 3*A*). Concomitantly, lower pFoxo1 localization in the cytosol was also evident in the *Smo*-KOs (Figure 3*B*). Consistent with this, expression of Foxo1 and several of its target gene mRNA transcripts (eg, *Pck1*, *Foxo1*, *Igfbp1*) were increased in *Smo*-KO livers, suggesting that loss of *Smo* impaired insulin signaling and alleviated the insulin-dependent suppression of gluconeogenesis (Figure 3*C*). To verify that *Smo* deletion impaired insulin action, insulin tolerance testing was performed in additional *Smo*-KO mice and vehicle-treated control mice. Consistent with insulin signaling data (Figure 2), insulin acutely decreased blood glucose in control mice, but this response was significantly impaired in *Smo*-KO mice (Figure 3*D*). Gene Ontology (GO) analysis of bulk RNA-seq data from additional mice demonstrated that insulin and insulin-like growth factor signaling and glucose homeostasis were significantly downregulated in the *Smo*-KO groups (Figure 3*E*), further confirming that disruption of insulin-mediated regulation of systemic glucose homeostasis is a consistent consequence of hepatocyte *Smo* deletion.

**Figure 4:**
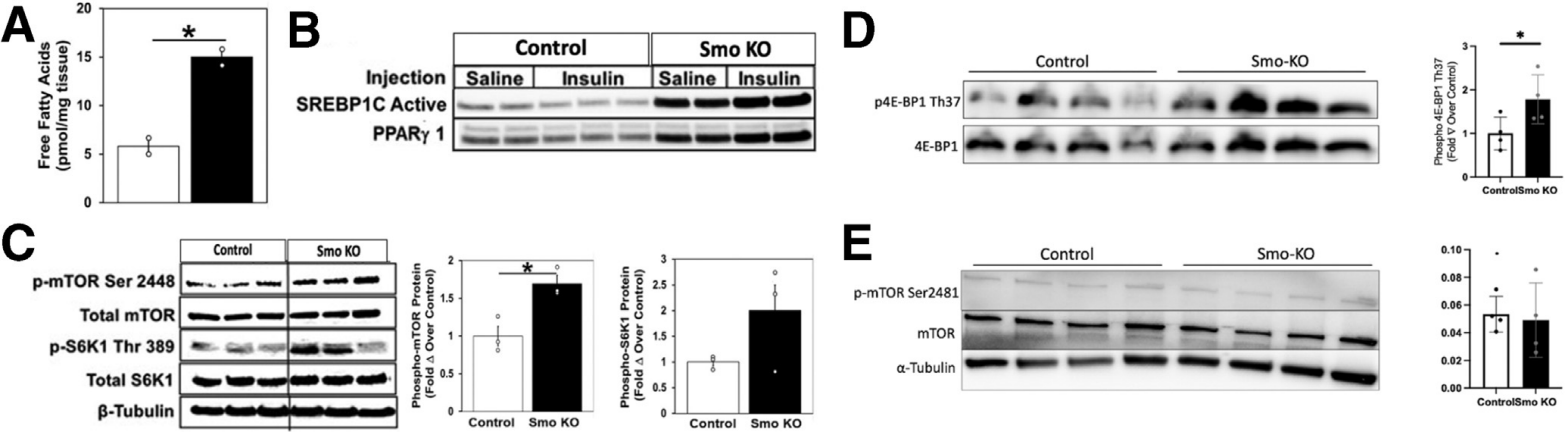
***Smo* deletion promote lipogenesis by activating mTOR.** (*A*) Hepatic free fatty acids (pmol/mg) in control and *Smo*-KO mice. (*B*) Protein immunoblots of SREBP1c and peroxisome proliferator-activated receptor (PPARγ1) in control and *Smo*-KO mice treated with saline or insulin. (*C*, *D*) Protein immunoblots of pmTOR Ser 2448 and its downstream effectors pS6K1 and p4E-BP1 in control and *Smo*-KO mice. (*E*) Protein immunoblots of pmTOR Ser 2481 in control and *Smo*-KO mice. The error bar indicates SEM. ∗*P* ≤ .05; ∗∗ P≤.01.

Defective insulin signaling may divert ingested glucose to fuel lipogenic pathways.^25^ Indeed, hepatic accumulation of free fatty acids was increased significantly in *Smo*-KO hepatocytes (Figure 4*A*). Sterol regulatory element binding protein-1c (SREBP1c) and peroxisome proliferator-activated receptor (PPARγ1) are transcription factors known to be key regulators of lipogenesis.^20^^,^^26^ We observed a strong induction of both their protein levels in saline- and insulin-treated *Smo*-KO livers (Figure 4*B*), supporting an earlier report indicating that *Smo* deletion promotes SREBP1c-driven induction of lipogenic genes.^17^ To elucidate mechanisms underlying effects of *Smo*-KO, we focused on mammalian target of rapamycin (mTOR) signaling because mTORC1 activates SREBP1c.^27^ Previous studies by us and others have demonstrated that inhibiting *Smo* blocks activation of AMPK, a well-established inhibitor of mTORC1.^16^^,^^18^^,^^28^ Therefore, we asked whether mTOR signaling was affected by *Smo* deletion. Immunoblots showed *Smo*-KO hepatocytes had increased phosphorylation at mTOR-S2448. Consistent with resultant mTORC1 activation, phosphorylation of mTORC1’s downstream effector, S6K1, on tyrosine 389 (S6K1-T389), was also increased (Figure 4*C*). Phosphorylation of another mTORC1 substrate, 4E-BP1, was also increased in *Smo*-KO hepatocytes, providing strong evidence that mTORC1 signaling was activated (Figure 4*D*). In contrast, phosphorylation of mTORC2 (mTOR-pSer2481) was unchanged in *Smo*-KO mice (Figure 4*E*). In addition to promoting lipogenesis, activated mTORC1 can directly promote IRS1/2 degradation to induce insulin resistance.^29^ As noted previously, total protein levels of IRS1/2 were downregulated in *Smo*-KO hepatocytes (Figure 2*B*). Taken together, the results demonstrate that hepatocyte *Smo* activity is necessary both for insulin and IR signaling that inhibits hepatocyte gluconeogenesis and to prevent mTORC1 from promoting lipogenesis and interfering with insulin signaling downstream of the IR. Thus, disrupting hepatocyte *Smo* activity revealed a unifying hepatocyte-driven mechanism that may explain the paradoxical observation that steatosis and insulin resistance typically correlate in NAFLD patients.

### Single-Cell Analysis Reveals *Smo* Active Hepatocytes Are Localized in the Midlobular Region

**Figure 5:**
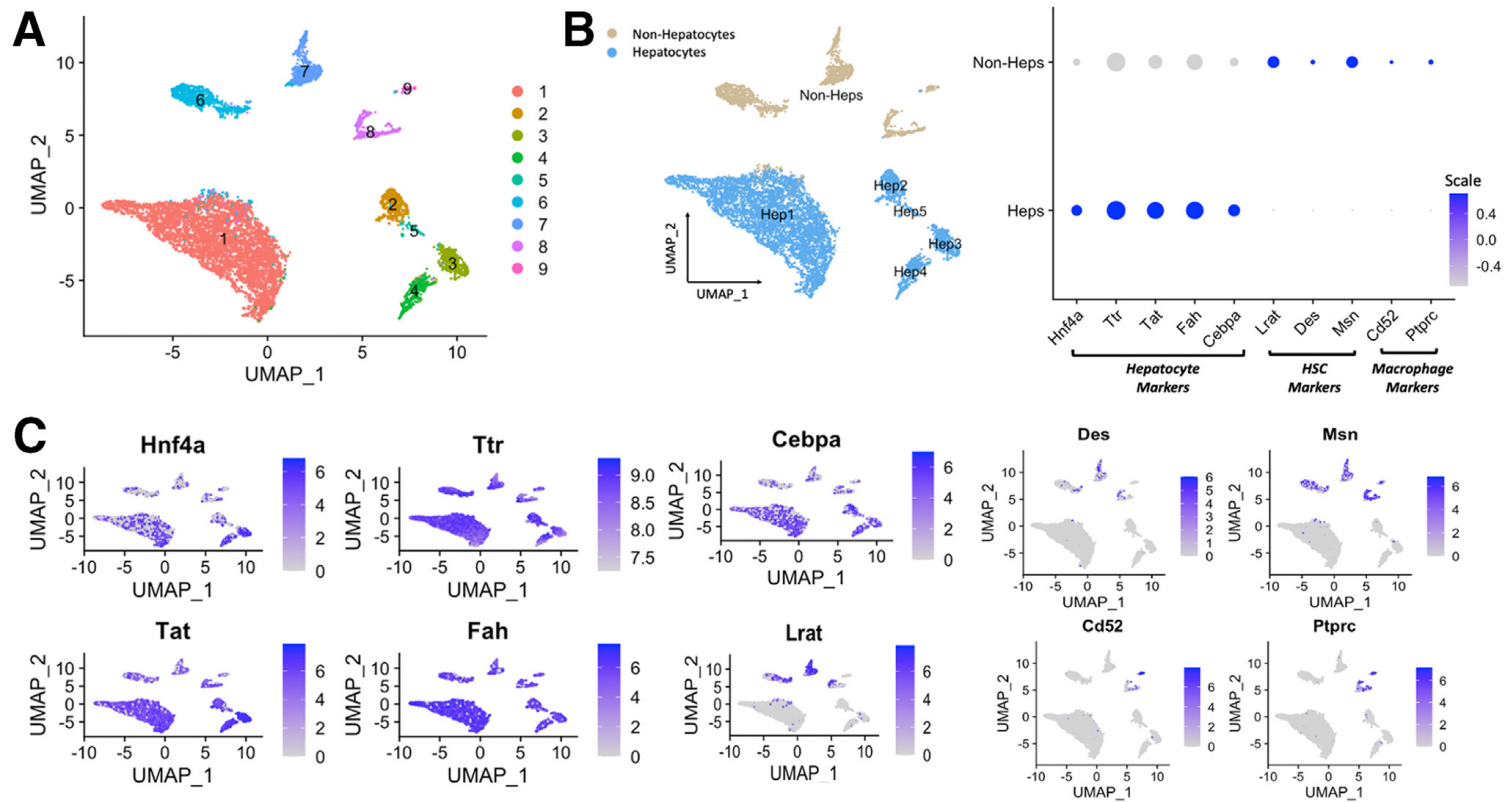
**Integrated single-cell analysis of control and *Smo*-KO hepatocytes.** (*A*) UMAP plot of all 9207 cells. (*B*) Cell type annotation. (*C*) UMAP plots of representative cell type–specific genes (eg, hepatocyte-specific markers), including *Hnf4a*, *Tfr*, *Tat*, *Fah*, and *Cebpa*; markers of hepatic stellate cells, including *Lrat*, *Des*, and *Msn*; and markers of Kupffer cells, including *Cd52* and *Ptprc*.

**Figure 6:**
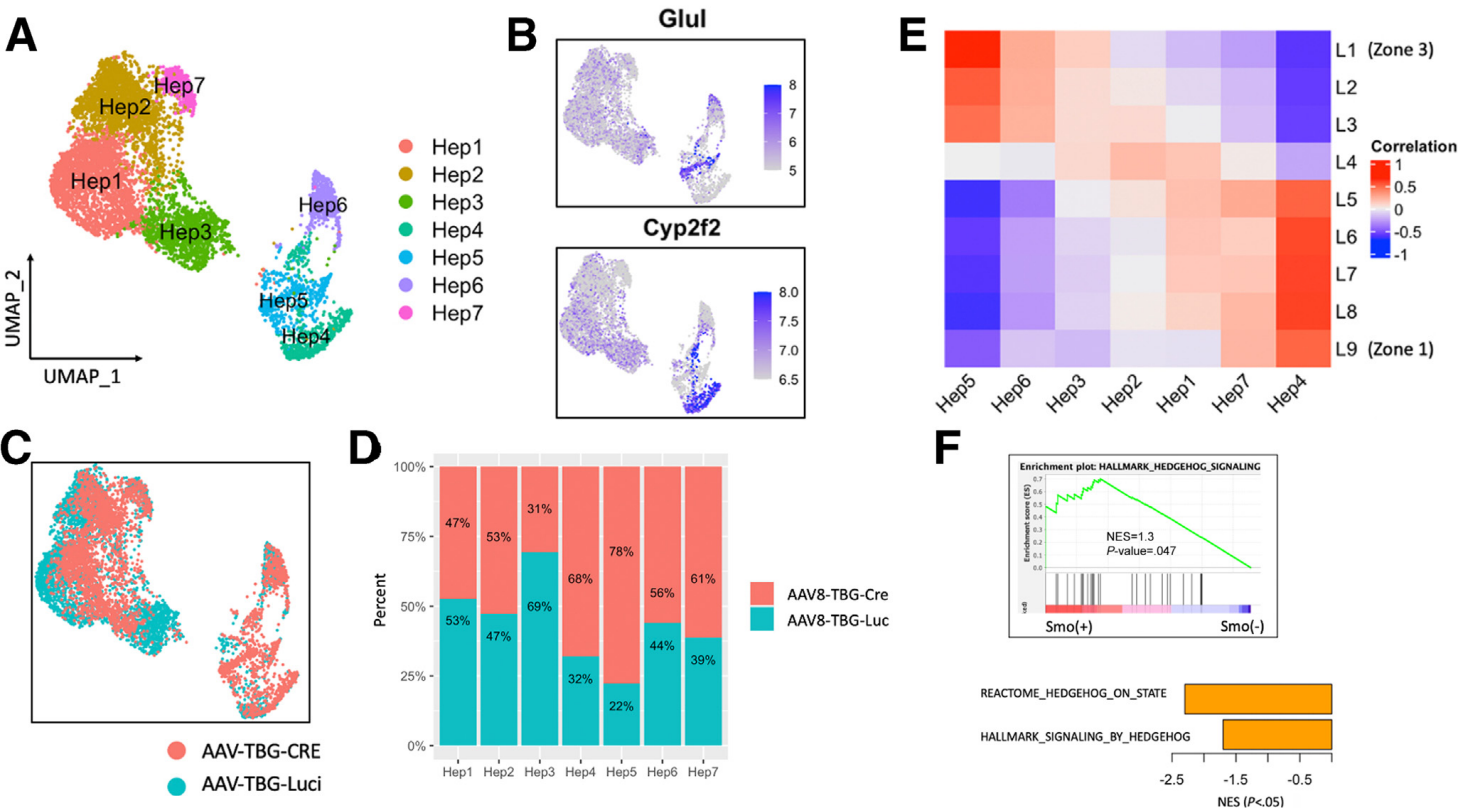
**Single-cell analysis reveals hepatocytes with *Smo* activity localize in midlobular region.** (*A*) UMAP plot of 6895 hepatocytes. (*B*) Expression of *Cyp2f2* and *Glul*. (*C*) Cell origin annotation. (*D*) Percent contribution of cells from AAV8-TBG-Luciferase (blue) and AAV8-TBG-Cre (red) treated mice in each hepatocyte cluster. (*E*) Heatmap showing correlation between *Smo* hepatocytes and zonal layers. (*F*) Enrichment analysis showing downregulation of Hedgehog pathway in *Smo*-KO hepatocytes.

The known heterogeneity of hepatocytes in healthy livers and the prominent role of *Smo* as a regulatory hub prompted us to seek further insight into its molecular interactions. For that, we performed single-cell RNA-seq on hepatocytes isolated from wild-type and *Smo*-KO mice. A total of 9207 cells were retained after passing through stringent quality control. Nine clusters were initially identified and annotated using putative liver cell markers (Figure 5*A*). Because the focus of this study was on hepatocytes, clusters 6–9 were excluded from subsequent analysis as they contained cells positive for nonhepatocyte markers (Figure 5*B* and *C*). Remaining hepatocytes were reclustered and analyzed (Figure 6*A*). Hep4 and Hep5 were differentially enriched for *Cyp2f2* and *Glul* and thereby were designated as periportal and pericentral hepatocytes, respectively (Figure 6*B*). Unlike Hep4/5/6/7, Hep1, Hep3, and to a lesser degree, Hep2 were affected by *Smo* deletion as exemplified by a marked shift in transcript expression between cells from each condition (Figure 6*C* and *D*). Correlation with established zonal layer signatures^30^ indicated that Hep1/2/3 were highly related to layers L3 to L7, suggesting that *Smo* active hepatocytes are localized in central-to-midzonal areas (Figure 6*E*). We were unable to reliably demonstrate *Smo* in healthy control mice using commercially available reagents for immunohistochemistry as our pilot immunoblot studies demonstrated multiple cross-reacting bands when these anti-*Smo* antibodies were used to detect *Smo* in whole liver extracts. Further, *Smo* is a low-abundance protein with a short half-life, and its activity in nonliver cells is strictly determined by its cellular localization.^31^, ^32^, ^33^ Therefore, we relied on GO and gene set enrichment analysis (GSEA) of bulk hepatocyte RNA-seq data and single-cell RNA-seq data to determine if Hedgehog pathway signaling was differentially active in *Smo*-KO and control mice. Both analyses demonstrated that the Hedgehog pathway activity was significantly suppressed in *Smo*-KO hepatocytes (Figure 6*F*), consistent with our earlier report that deleting *Smo* in hepatocytes reduced mRNA expression of Hedgehog target genes (eg, *Gli1* and *Ptch*).^18^

### *Smo* Single-Cell Analysis Identifies Mitochondrial Dysfunction and DNA Damage

**Figure 7:**
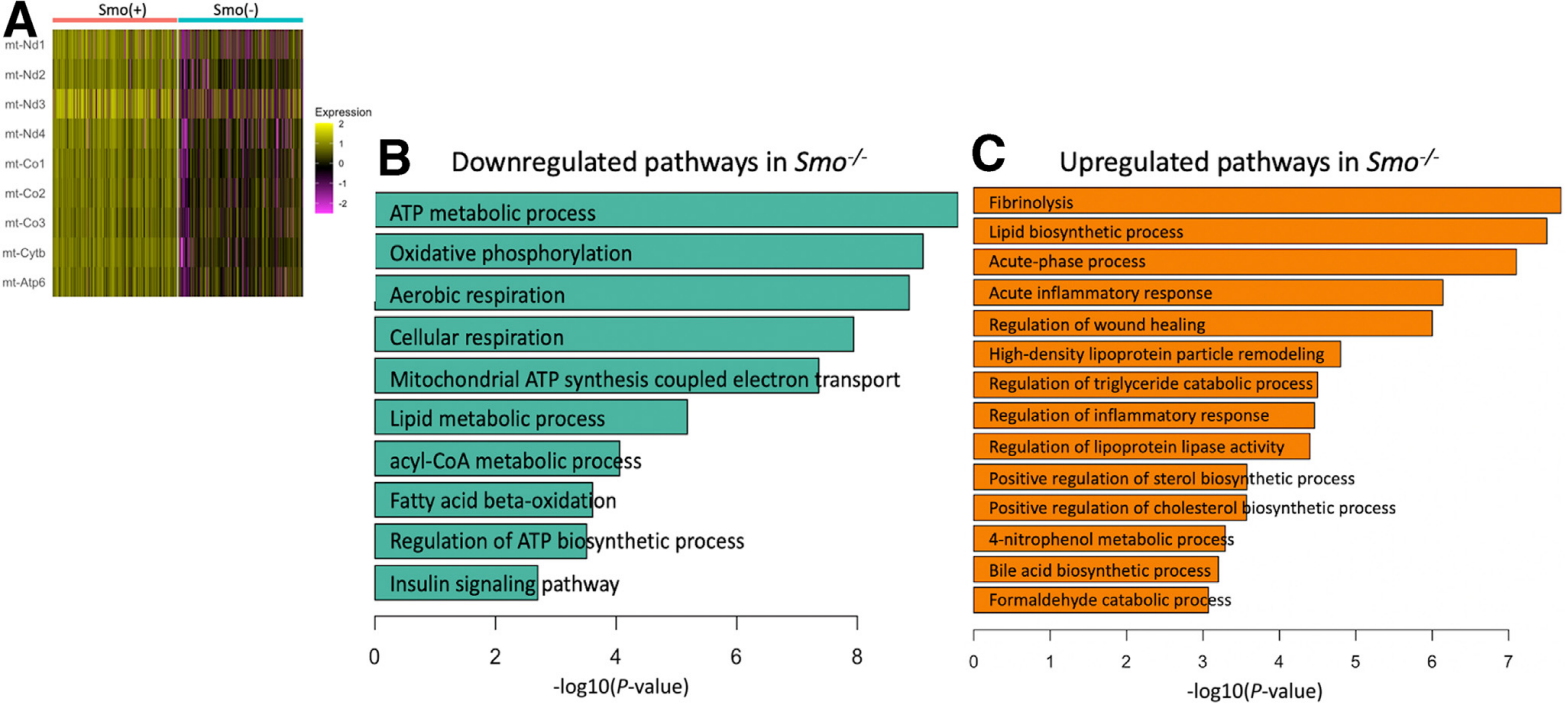
**Analysis of differentially expressed genes demonstrates pathways that are dysregulated after *Smo* deletion.** (*A*) Heatmap showing downregulation of mitochondrial genes in *Smo*-KO vs control hepatocytes. (*B*, *C*) Pathways that are significantly downregulated or upregulated in *Smo*-KO hepatocytes.

**Figure 8:**
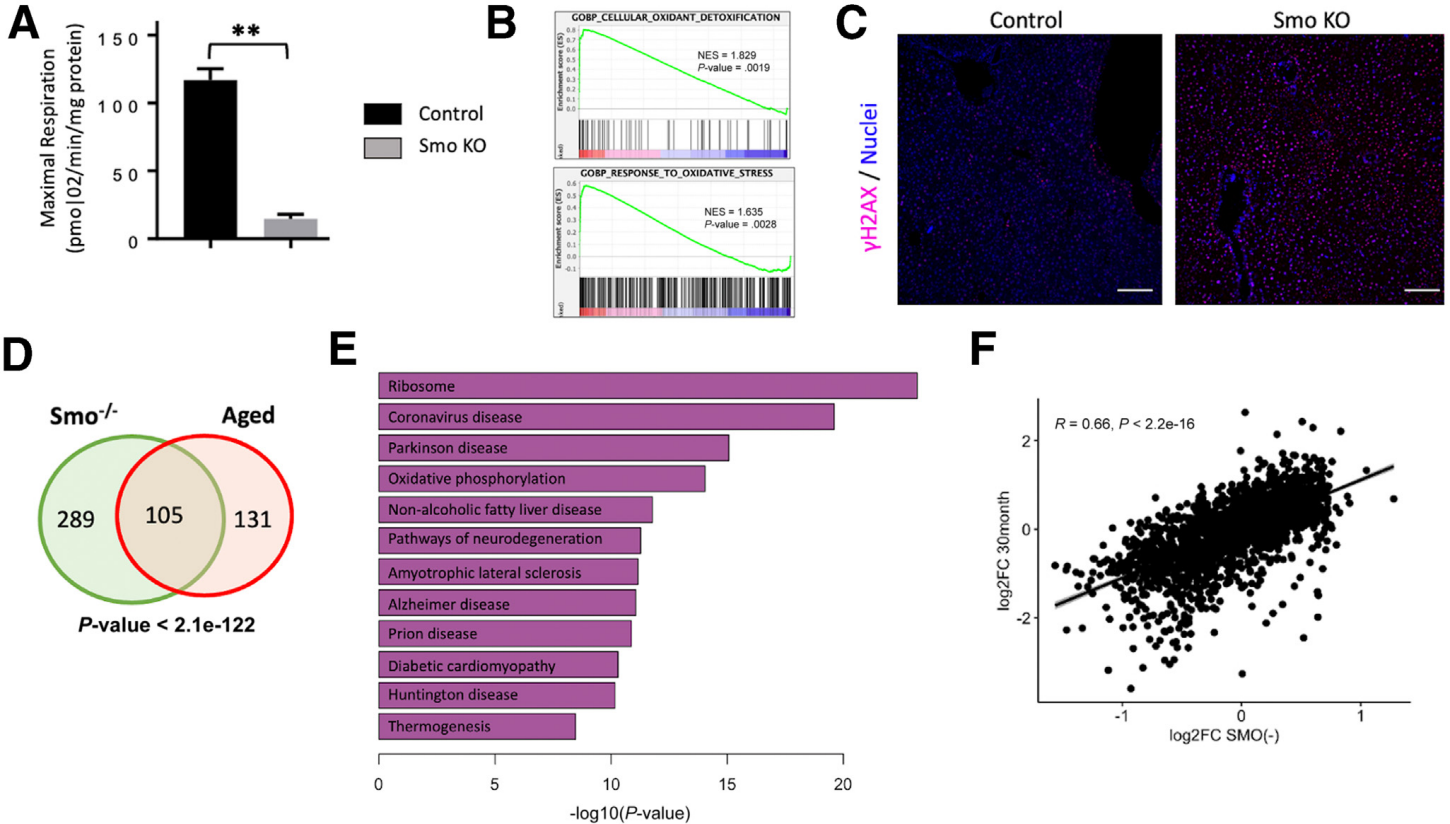
***Smo* deletion causes hepatocyte mitochondrial dysfunction and DNA damage.** (*A*) Seahorse studies demonstrate reduced hepatocellular respiration after *Smo* knockout. ∗∗*P* ≤ .005 by *t* test. (*B*) GSEA analysis of single-cell RNA-seq data from *Smo*^*–/–*^ vs *Smo*^*+/+*^ hepatocytes. (*C*) γH2AX staining in control and *Smo*-KO livers. Scale bars = 10 μm. (*D*) Comparison of genes differentially upregulated in *Smo*^*–/–*^ hepatocytes and aged hepatocytes vs their respective controls. Fisher’s exact test was used to assess significance of shared upregulated genes (ie, overlap). (*E*) Functional analysis demonstrates disease categories enriched in the overlapped genes. (*F*) Correlation between the 2 datasets as calculated using Pearson correlation: *r* = 0.66, *P <* 2.2 × 10^–16^.

Within the Hep1/3 clusters, analysis revealed that *Smo*^*+/+*^ and *Smo*^*–/–*^ hepatocytes differed in gene expression and pathway activities (Figure 7*A*–*C*). For example, *Smo*^*–/–*^ cells exhibited downregulation of pathways linked to homeostasis of lipid, fatty acid beta-oxidation, and insulin signaling, which agreed with our mouse liver data. Interestingly, *Smo*^*–/–*^ hepatocytes also downregulated biological processes such as cellular respiration, oxidative phosphorylation, and ATP synthesis, implying mitochondrial malfunction. Indeed, mitochondrial genes encoding components of the electron transport chain and ATP synthase were among the most downregulated genes in the *Smo*^–^ compared with the *Smo*^+^ cluster, suggesting that *Smo* deletion impairs mitochondrial function in hepatocytes. The significance of these observations was confirmed by an experiment in which primary hepatocytes from control mice and *Smo*-KO mice were cultured for 24 hours and then their mitochondrial bioenergetics were surveyed using a Seahorse analyzer (Agilent, Santa Clara, CA). *Smo*-depleted hepatocytes demonstrated markedly suppressed respiratory capacity (Figure 8*A*), consistent with our previous study which showed that treating cultures of wild-type primary hepatocytes with cyclopamine, a *Smo* inhibitor, significantly reduced their mitochondrial oxygen consumption and ATP production.^34^

We reasoned that this mitochondrial dysfunction may contribute to lipotoxicity. Mitochondrial dysfunction is known to promote oxidative stress, which may lead to DNA damage.^35^ As expected from transcriptional data, the single-cell analysis also demonstrated significant upregulation of oxidant detoxification pathways and response to oxidative stress in *Smo*^*–/–*^ hepatocytes (Figure 8*B*). Moreover, fluorescent imaging showed enhanced staining for DNA damage in *Smo*-KO livers (Figure 8*C*). Many of the abnormalities triggered by *Smo* deletion are hallmarks of aging,^36^^,^^37^ and we recently reported that Hedgehog was a top downregulated pathway in hepatocytes isolated from very old mice.^34^ To determine whether *Smo*^*–/–*^ hepatocytes display similar transcriptional changes as aged animals, we compared gene expression of *Smo*^*–/–*^ hepatocytes with that of a published dataset derived by others from a 30-month-old mouse liver (GSE132042).^38^ Significant overlap of differentially upregulated genes between the 2 datasets was observed (*P* < 2.1 × 10^–122^) (Figure 8*D*). Functional analysis of those overlapped genes revealed significant enrichment of GO terms related to tissue degeneration (eg, Parkinson’s disease and Huntington’s disease) (Figure 8*E*). Global gene expressions were also strongly concordant (*P* < 2.2 × 10^–16^ and R = 0.66) (Figure 8*F*). Together, these findings indicate that loss of *Smo* induced premature aging in hepatocytes, promoted accumulation of metabolically defective stressed hepatocytes, and activated gene programs that are typical of degenerative diseases.

### *Smo*-KO Hepatocytes Regulate Proinflammatory and Wound Healing Responses

NAFLD was one of the most mutually enriched GO terms in *Smo*-depleted young mouse hepatocytes and livers of very old mice (Figure 8*E*), further supporting a role for premature hepatocyte aging in NAFLD pathogenesis. Stressed cells that accumulate in aging tissues express secretomes that promote inflammation and fibrosis,^39^^,^^40^ processes that are involved in NAFLD progression.^41^ GO analysis of our hepatocyte single-cell RNA-seq data demonstrated significant enrichment for transcripts involved in acute phase processes, acute inflammatory response, and regulation of wound healing in *Smo*^–/–^ vs *Smo*^+/+^ hepatocytes (Figure 7*B*). These hepatocyte transcriptional responses were evident within a week of hepatocyte-specific deletion of *Smo*, a time point when *Smo*-depleted livers demonstrated significant steatosis but relatively mild aminotransferase elevations (Figure 1*B* and *F*). Thus, lipotoxic stress imposed by short-term loss of hepatocyte metabolic flexibility might be sufficient to initiate progression from hepatic steatosis (NAFL) to NASH by promoting maladaptive inflammatory repair responses that, when chronic, eventuate in cirrhosis.

### *Smo* Expression Inversely Correlates With NAFLD Severity in Humans

**Figure 9:**
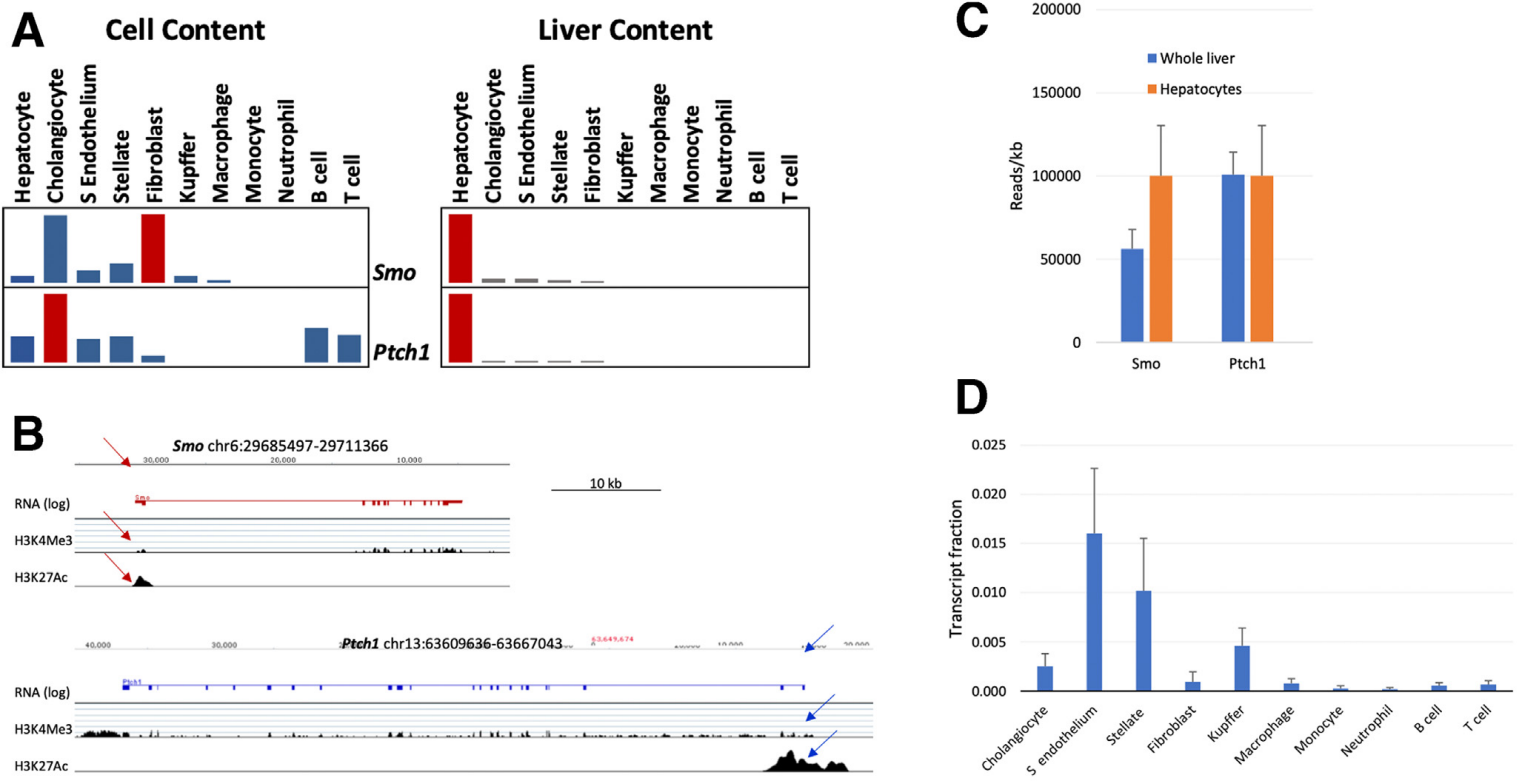
**Cell type–specific expression of *Smo* and *Ptch1* in mouse livers.** (*A*) Comparison of expression in each cell type. The genes were compared using a normalized set of mouse RNA-seq libraries representing 11 liver cell types (unpublished data, J. Locker, MD, November 2022). (Left) *Smo* is most strongly expressed in cholangiocytes, fibroblasts, and stellate cells but is also detected in sinusoidal endothelium and hepatocytes. *Ptch1* expression is strongest in cholangiocytes, but is detected in hepatocytes, sinusoidal endothelium, stellate cells, B cells, and T cells. In this display, the transcript levels have been normalized by the median values of each RNA-seq library, but because the size of cell-type transcriptomes may be different, any of the detected levels might be significant. (Right) The plot displays the fractional contribution of each cell type to total liver expression and shows that almost all the *Smo* and *Ptch1* transcripts come from hepatocytes, which are calculated to provide 96% of total liver cell transcripts. Calculated proportions of other cell-type transcriptomes in normal liver: cholangiocyte = 0.3%; stellate cell = 1.0%, sinusoidal endothelium = 1.6%, and fibroblast = 0.1%. Each plot is normalized to the maximum expression level. From primary libraries, a set of cell type–specific transcripts was compiled, and for each transcript within the set the following ratio was calculated whole liver transcriptome fraction / cell-type transcriptome fraction. The average of values for multiple transcripts approximated the fraction of the cell-specific transcriptome within the total liver transcriptome. (*B*) RNA-seq and chromatin immunoprecipitation sequencing visualizations of *Smo* and *Ptch1* in normal liver. Despite relatively low levels, transcripts are clearly detected. The detection of H3K4Me3 (active promoters) and H3K27Ac (transcriptionally active chromatin) indicates that the promoters (arrows) are active in hepatocytes. The datasets are described in reference 113 RNA-seq and chromatin immunoprecipitation sequencing are displayed on log and linear scales, respectively. (*C*) *Smo* and *Ptch1* co-purify with hepatocytes. The increased expression of *Smo* suggests that expression is stimulated by isolation, a common reactive phenotype. RNA-seq data were the following: n = 2 for each group; plots show mean ± SD; transcripts are quantified as reads/kb. (*D*) Cell-type fractions of the total liver transcriptome. The ratios (t_L_/t_c_) for multiple cell type–specific genes were averaged for each cell type. The plot shows mean ± SD.

**Figure 10:**
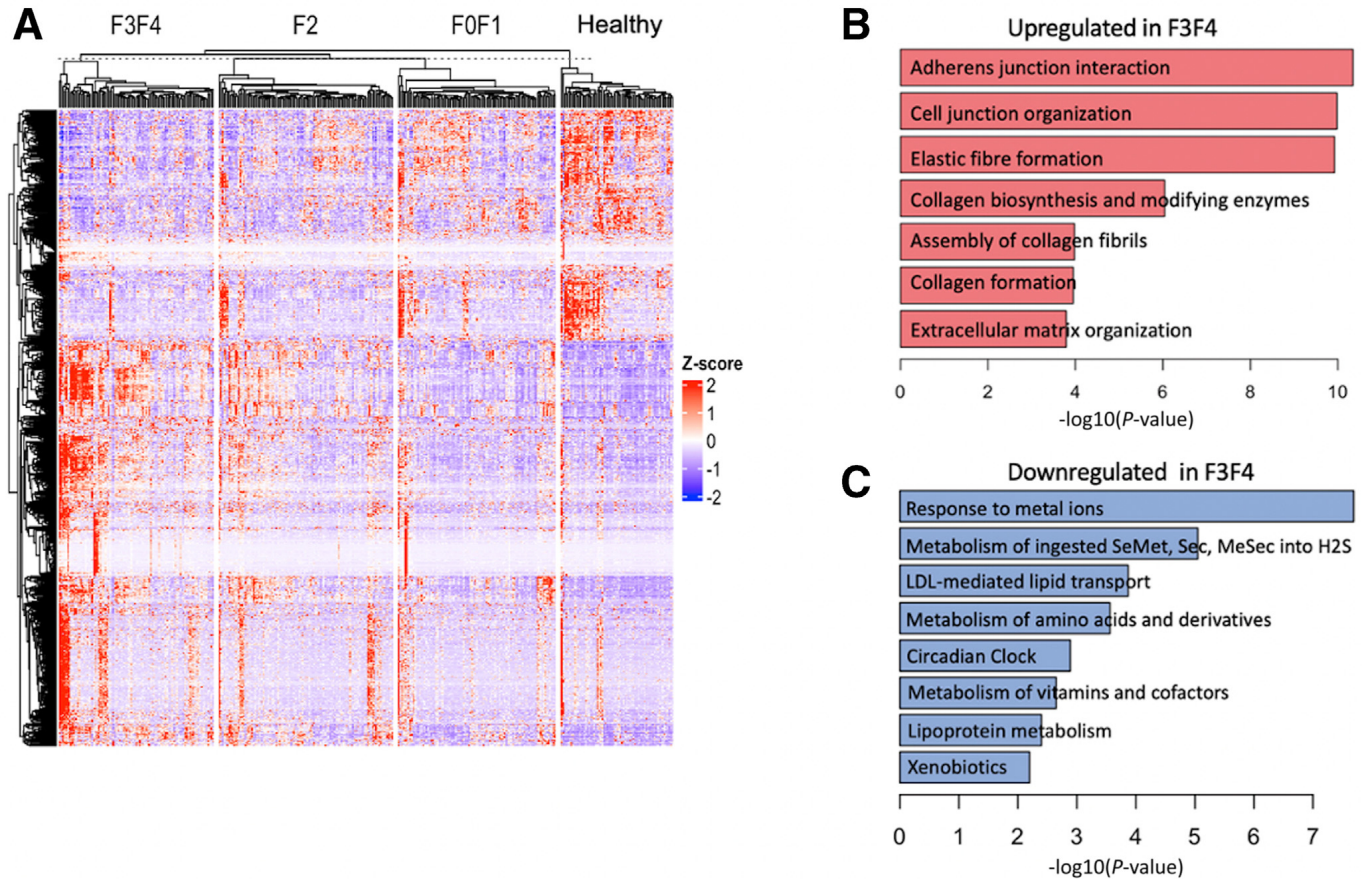
**Differential gene expression analysis of human liver samples with or without NAFLD.** Liver samples from subjects undergoing evaluation for suspected NAFLD grouped according to histologic diagnosis (normal/nonspecific changes [n = 69] or NAFLD with little to no fibrosis [F0/F1], early bridging fibrosis [F2], and advanced fibrosis [F3/F4] [n = 299]). (*A*) Heatmap showing genes differentially regulated between groups. (*B*, *C*) Pathways that are upregulated or downregulated in NAFLD livers with advanced (F3/F4) fibrosis vs livers without NAFLD (healthy control subjects). LDL, low-density lipoprotein.

**Figure 11:**
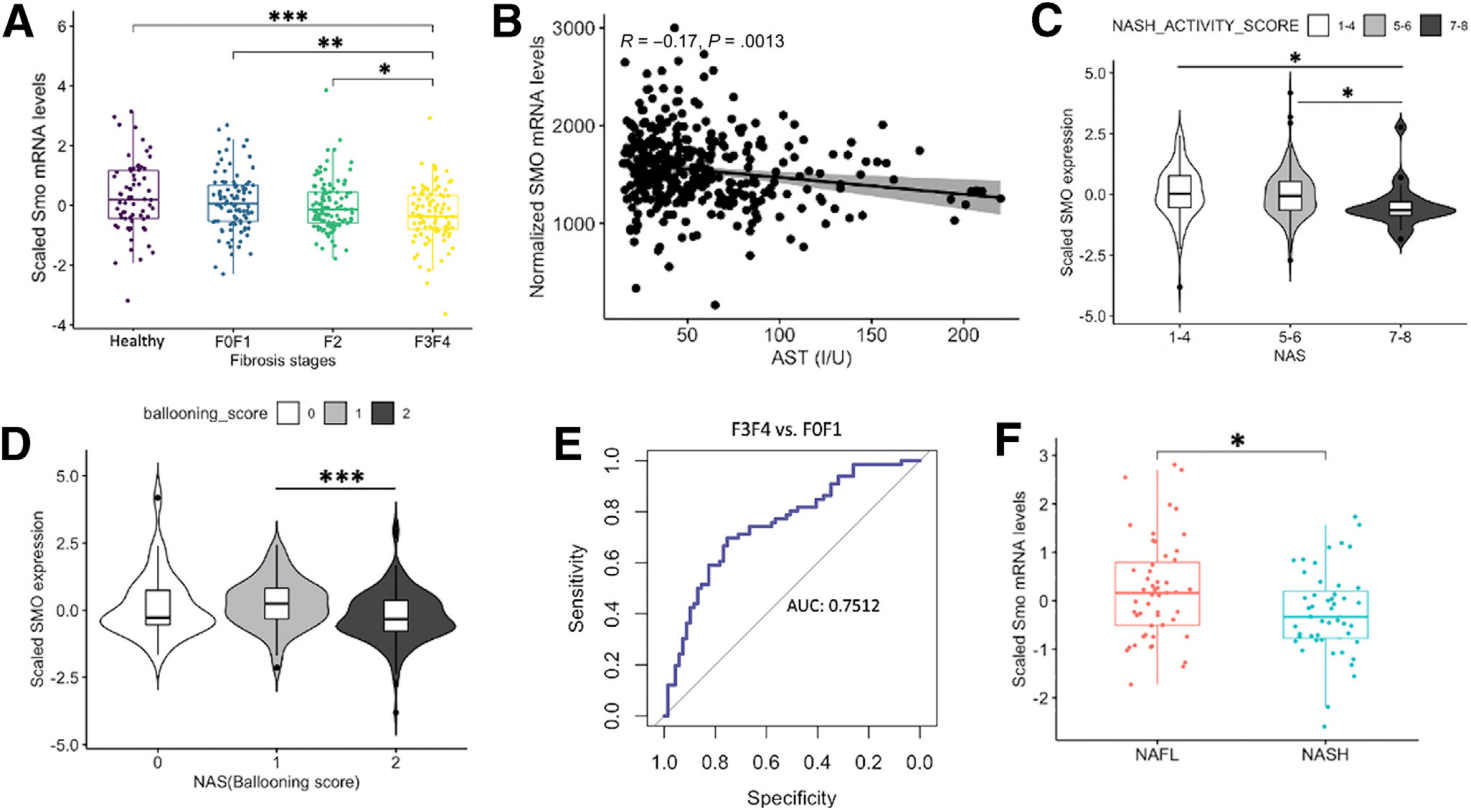
***Smo* expression and NAFLD severity are inversely correlated.** Further analysis of patient cohort described in Figure 9. (*A*) Boxplot of liver *Smo* expression according to NAFLD fibrosis stage. (*B*) Correlation between *Smo* mRNA expression and serum AST. (*C*, *D*) *Smo* mRNA levels vary with NASH activity score and ballooning scores. (*E*) *Smo* transcript levels distinguish NAFLD subjects with advanced (F3/F4) fibrosis from those with mild (F0/F1) fibrosis. Area under the curve is shown. (*F*) Boxplot of liver *Smo* expression in an independent cohort of NAFLD patients (see text for details). The error bar indicates SEM. ∗*P* ≤ .05; ∗∗*P* ≤ .005; ∗∗∗*P* ≤ .001.

To investigate the physiological relevance of *Smo* in humans, we leveraged available bulk human liver RNA-seq datasets acquired from biopsies of almost 500 unique subjects. Pilot studies were done in bulk RNA-seq libraries from mouse livers and various mouse liver cell subpopulations to determine the relative abundance of *Smo* and *Ptch*, the 2 cell surface receptors that are required for Hedgehog ligand signaling. The results demonstrated that most of the *Smo* and *Ptch* transcripts in whole liver RNA derive from hepatocytes, although hepatocyte expression of these genes is low on a per cell basis (Figure 9*A–D*). Thus, disease-related changes in levels of *Smo* transcripts in the bulk liver RNA-seq datasets will be dominated by changes in *Smo* expression of hepatocytes (the predominant cell population), despite accumulation of stromal cell types that express higher levels of *Smo* mRNA on a per-cell basis. Confident that bulk RNA-seq analysis could capture changes in hepatocyte *Smo* expression, we performed high-throughput RNA-seq on liver samples collected from 368 patients suspected of having NASH fibrosis who underwent a diagnostic liver biopsy. Subjects with and without NAFLD were comparably obese and did not differ significantly in age. However, NALFD subjects were more likely to have a diagnosis of type 2 diabetes (47.8% vs 17.4%; *P <* 0.0001) (Table 2). We stratified the patients into 4 groups based on liver histology (eg, healthy or NAFLD with F0/F1, F2, or F3/F4 fibrosis) and analyzed differentially expressed genes to identify transcriptional changes that occurred across this spectrum of liver damage (Figure 10*A*). As expected, analysis of upregulated genes in NAFLD livers with F3/F4 fibrosis revealed enrichment of pathways associated with collagen biosynthesis and extracellular matrix organization, whereas pathways related to liver functions were suppressed (Figure 10*B* and *C*). Notably, *Smo* expression showed a stepwise downregulation such that its expression was highest in the healthy-appearing livers and declined progressively in NAFLD as fibrosis stage worsened (Figure 11*A*). Furthermore, *Smo* negatively correlated with serum AST, NASH activity score, and hepatocyte ballooning scores (Figure 11*B–D*), linking *Smo* loss to increased severity of lipotoxic liver injury. Indeed, *Smo* expression could differentiate advanced NAFLD from mild NAFLD with reasonable accuracy (area under the curve, 0.75) (Figure 11*E*). Analysis of a dataset from an independent Japanese NAFLD cohort (GSE167523)^42^ also showed that *Smo* expression is lower in NASH than NAFL (Figure 11*F*). Together, these results complemented our findings from mice and supported the concept that *Smo* critically regulates susceptibility to NAFL, NASH, and NASH-related fibrosis.

**Table 2:** Patient Characteristics

	Control Cohort (n = 69)	NAFLD Cohort (n = 299)	*P* Value
Female	81%	61	.013
Race			
White	72.5	84.9	.02
Black	21.7	8.7	
Asian	2.9	2.0	
Hispanic	0.0	1.0	
American Indian	0.0	0.7	
Multiple	2.9	1.0	
Unknown	0	1.7	
Age, y	47.4 ± 12.6	50.5 ± 12.8	.07
BMI, kg/m^2^	37.4 ± 8.2	36.8 ± 8.1	.58
Diabetes	17.4	47.8	*<.0001*
Hyperlipidemia	46.4	34.4	.07
Hypertension	43.5	32.4	.17
Fibrosis stage			
0	100.0	10.0	*<.0001*
1	—	22.4	
2	—	35.8	
3	—	27.1	
4	—	4.7	
NASH score			
≤3	—	1.7	
4	—	35.5	
5	—	36.5	
6	—	18.7	
≥7	—	7.7	

Values are % or mean ± SD.

BMI, body mass index; NAFLD, nonalcoholic fatty liver disease; NASH, nonalcoholic steatohepatitis.

**Figure 12:**
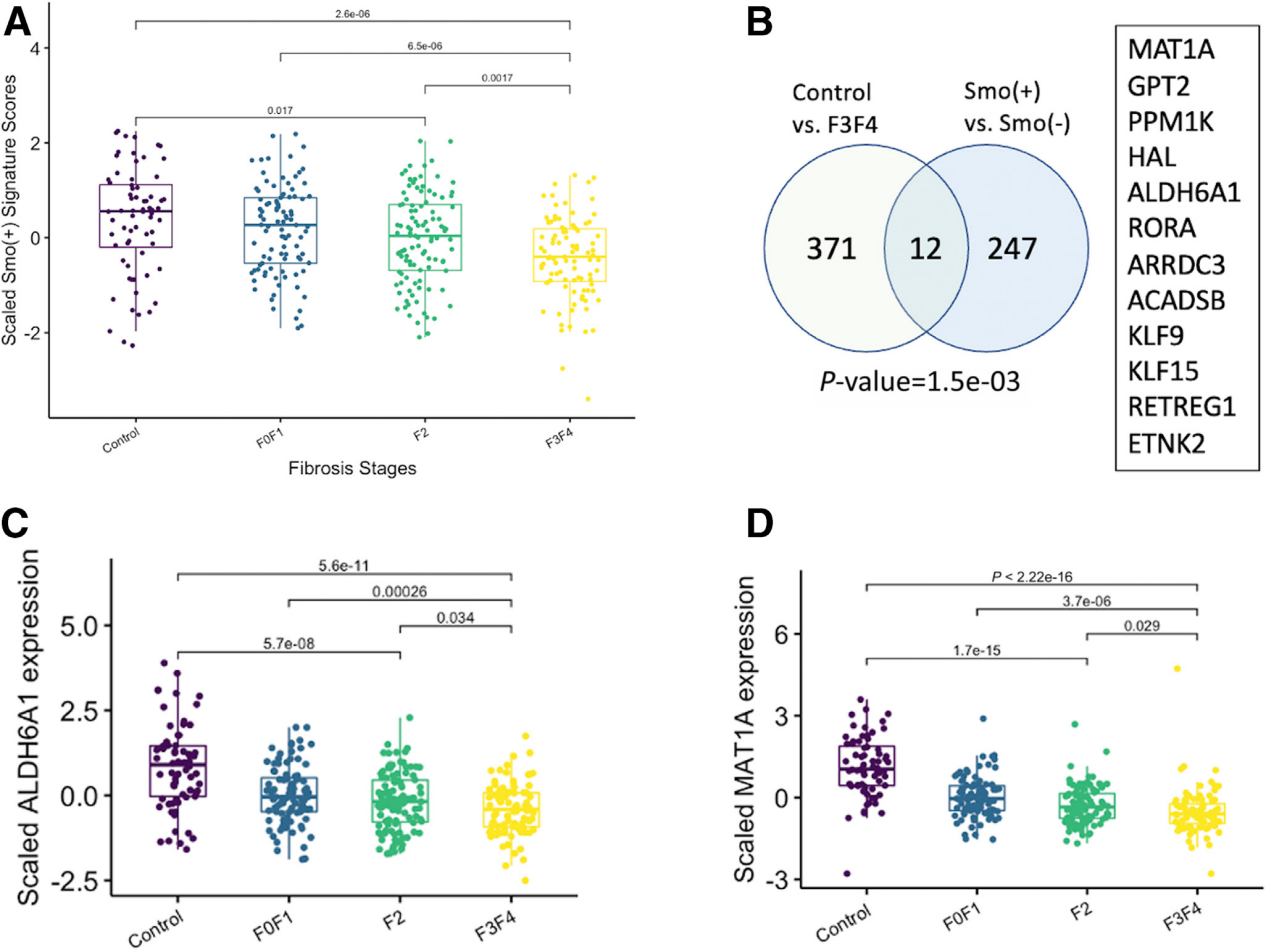
**Analysis of mouse-derived *Smo* positive signature in human NAFLD data.** (*A*) A *Smo*^+^ gene signature score was generated by analyzing single-cell RNA-seq data to identify genes that were significantly upregulate healthy control (*Smo*^+/+^) vs *Smo*-deleted (*Smo*^–/–^) mouse hepatocytes. Boxplot showing the estimated scores of *Smo*^+^ gene signature in patients with healthy-appearing livers (control subjects) and patients with different fibrosis stages of NAFLD. (*B*) Venn diagram displaying shared gene set that is upregulated in the mouse hepatocyte *Smo*^+^ signature and healthy human livers vs *Smo*-depleted hepatocytes and human livers with advanced (F3/F4) NAFLD fibrosis; gene overlap significance was determined using Fisher’s exact test. (*C*, *D*) Boxplot of *ALDH6A1* and *MAT1A* expressions in NAFLD cohort.

Hepatocyte-specific deletion of *Smo* caused hepatic steatosis (Figure 1), insulin resistance (Figure 2), and lipotoxicity (Figure 8) in our mouse model; single-cell analysis linked these liver phenotypes with dynamic changes in hepatocyte gene expression and pathway activities that occurred in *Smo*-KO hepatocytes with inhibited Hedgehog signaling (Figures 6 and 7). To evaluate the functional significance of *Smo* activity in human hepatocytes, we used our mouse single-cell RNA-seq data to generate a transcriptional signature composed of transcripts most significantly upregulated in luciferase-treated (vehicle-treated control) vs Cre-treated (*Smo*-KO) hepatocyte populations. This signature gene set was then applied to the human bulk RNA-seq data from the cohort of 368 patients. We found that the activity of this signature gene set paralleled that of *Smo* mRNA expression in the patient cohort (ie, the calculated score was highest in the healthy-appearing control livers); among the NAFLD cohort, it declined progressively as the severity of liver fibrosis increased (Figure 12*A*). Further analysis identified a small subset of genes that were upregulated in both the single-cell RNA-seq–derived signature and bulk RNA-seq data from the healthy control human livers relative to the human livers with advanced NASH fibrosis (Figure 12*B-D*). Although only 1 of these genes had been linked to the Hedgehog pathway previously, remarkably, each encodes a factor that has been shown to exert protective effects that impede evolution of NAFLD or MetS.^43^, ^44^, ^45^, ^46^, ^47^, ^48^, ^49^, ^50^, ^51^, ^52^, ^53^, ^54^, ^55^, ^56^, ^57^, ^58^, ^59^, ^60^, ^61^, ^62^, ^63^, ^64^, ^65^, ^66^, ^67^, ^68^, ^69^, ^70^, ^71^, ^72^, ^73^, ^74^, ^75^, ^76^ For example, hepatocytes with *Smo* activity express significantly higher levels of MAT1-A and ALDH6A1 than *Smo*-KO hepatocytes. MAT1-A controls synthesis of S-adenosyl methionine and thus, glutathione homeostasis and vulnerability to oxidant stress. Livers of MAT1A-KO mice become depleted of reduced glutathione and spontaneously develop fibrosing NASH and liver cancer.^43^ Our previously published microarray analysis of humans with NAFLD showed that MAT1A expression is suppressed in livers with advanced vs mild NAFLD fibrosis.^44^ Roughly half (49%) of humans with NAFLD in an independent cohort were found to have metabolomic signatures that were similar to those of MAT1A-knockout mice.^45^ *ALDH6A1* is a mitochondrial gene that is highly expressed in normal liver where it regulates mitochondrial function, including reactive oxygen species generation. *ALDH6A1* is decreased in aging and loss of hepatic *ALDH6A1* is thought to compromise quality-control mechanisms that promote the apoptosis of hepatocytes with dysfunctional mitochondria.^55^ Collectively, these findings reveal a previously unsuspected role for hepatocyte Hedgehog signaling in regulating susceptibility to human NAFLD and suggest that inhibition of this signaling pathway promotes lipotoxicity and thus may exacerbate NAFLD progression.

## Discussion

NASH is the liver correlate of MetS, an insulin-resistant proinflammatory state that promotes progressive degeneration of vital organs, including the liver.^1^ Although the root cause of the MetS is uncertain, the condition is characterized by dysregulated lipid and glucose metabolism and thus, organ degeneration is thought to result from chronic metabolic stress.^77^ Development of effective pharmacologic approaches to prevent or treat NASH has been limited by poor understanding of fundamental mechanisms that determine hepatocyte resiliency, including the ability to adapt to—and recover from—metabolic stress.

Herein we provide novel evidence that basal metabolic homeostasis in adult hepatocytes is controlled by *Smo*, an obligate component of the Hedgehog signaling pathway that orchestrates fate decisions in liver progenitors during development. Remarkably, the new data reveal that without *Smo*, adult hepatocytes in otherwise healthy mice struggle to maintain metabolic flexibility and rapidly develop insulin resistance and lipotoxicity, even without superimposition of exogenous obesogenic stressors that are thought to drive MetS pathogenesis and end-organ damage in humans. Our results also challenge assumptions about human NAFLD pathogenesis by revealing that hepatic *Smo* activity and fatty liver pathology are inversely related in obese humans. More specifically, we found that hepatic *Smo* activity is greater in obese patients with healthy-appearing livers than in comparably obese patients with NAFLD, and demonstrated that *Smo* declines in a stepwise fashion as the severity of liver injury and fibrosis increase. The human data also revealed associations between liver *Smo* activity and type 2 diabetes, supporting our preclinical work which showed that deleting *Smo* in hepatocytes is sufficient to cause insulin resistance. To our knowledge, this is the first report that establishes a connection between Hedgehog pathway disruption in hepatocytes, hepatic insulin resistance, and both pathogenesis and progression of NAFLD.

**Figure 13:**
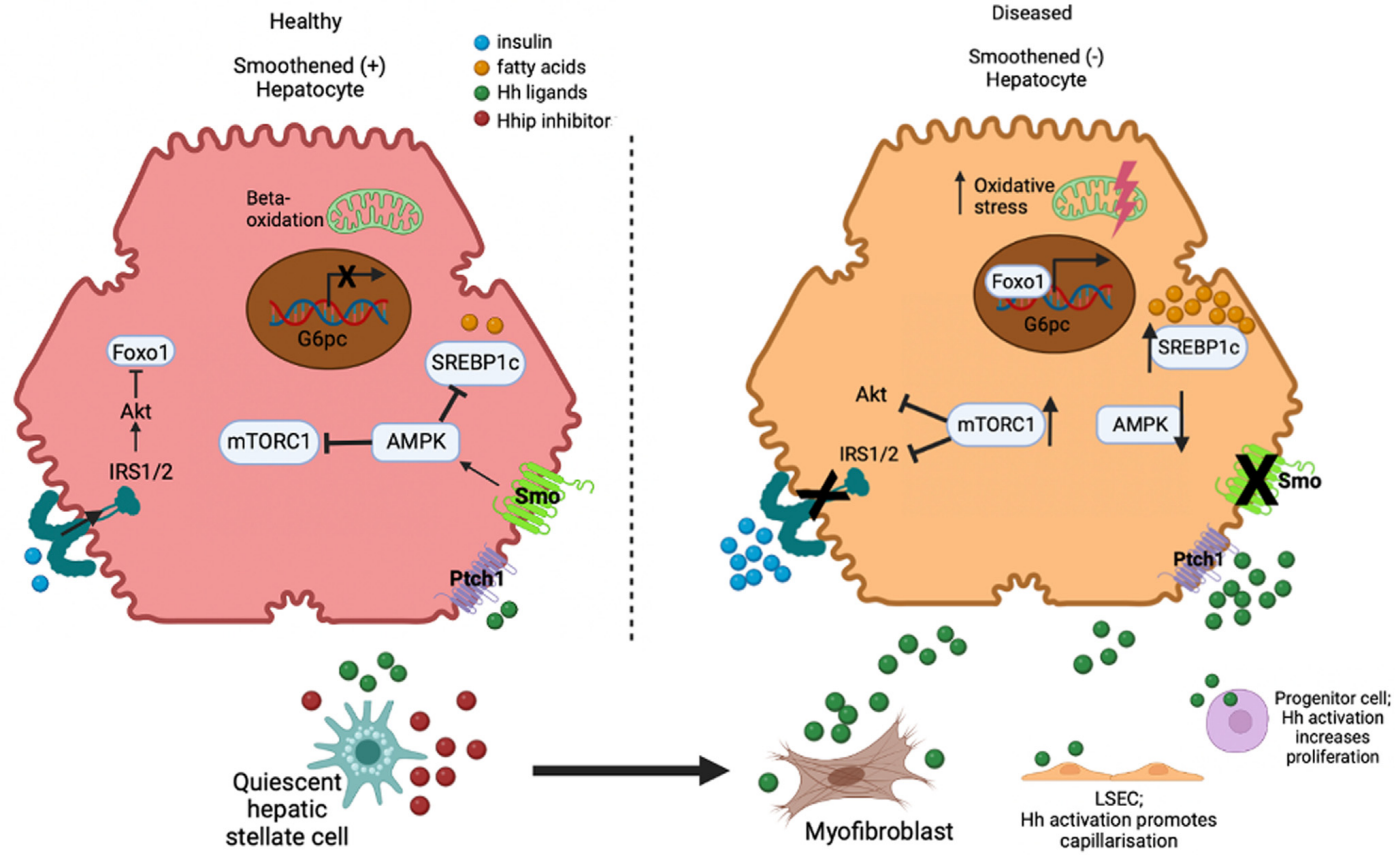
**Model for *Smo*-mediated protection from steatosis and lipotoxicity.** In healthy livers (left), subpopulations of hepatocytes that express essential Hedgehog (Hh) pathway signaling components (eg, *Ptch* and *Smo*) are exposed to Hh ligands that are released from neighboring cells (eg, hepatic stellate cells) and escape the soluble Hh ligand inhibitors (eg, Hhip) that their neighbors also produce. These “free” Hh ligands interact with *Ptch* on the surface of hepatocytes and this permits activation of *Smo* to initiate intracellular signaling cascades, one of which leads to the phosphorylation/activation of AMPK. Activated AMPK, in turn, restricts activation of mTORC1 (a kinase that can inhibit both insulin signaling and autophagy). High AMPK activity or low mTORC1 activity limits accumulation of SREBP1c (a lipogenic transcription factor) and supports autophagy of both lipid droplets and damaged mitochondria. Hence, hepatocytes that can activate *Smo* are sensitive to insulin inhibition of gluconeogenesis and do not become fatty because they have little de novo lipogenesis, are capable of lipophagy, and are enriched with healthy mitochondria that can oxidize fatty acids. In diseased livers (right), stromal cells downregulate their production of Hhip and stressed cells upregulate their production of Hh ligands. All types of liver cells that retain the ability to activate *Smo* remain sensitive to insulin (a factor that promotes cell viability and proliferation) and are protected from lipotoxcity, enabling their outgrowth and thereby dynamically reconfiguring the hepatic microenvironment to support eventual repopulation of the liver with healthy hepatocytes. However, this healthy regenerative process is not possible if the hepatocyte compartment is unable to activate *Smo* because *Smo*-deficient hepatocytes struggle to activate AMPK and thus, exhibit overactivation of mTORC1 which reduces their sensitivity to insulin while impairing clearance of damaged mitochondria and promoting synthesis of lipid substrates that can be oxidized. In aggregate, these responses enhance hepatocyte vulnerability to oxidative stress and lipotoxicity that results from peroxidation of cellular lipids. Lipotoxicity is a dynamic process that can be catalyzed by iron and prevented or reversed by factors that limit production of H_2_O_2_, constrain accumulation of lipid targets, or enhance the activity of enzymes that detoxify lipid peroxides. Thus, *Smo*-deficient cells that experience lipotoxic stress can undergo adaptations that permit their recovery or at least, delay their ultimate demise. The latter response leads to accumulation of *Smo*-deficient hepatocytes and these metabolically stressed hepatocytes that are no longer able to regenerate amplify their production of Hedgehog ligands and other paracrine wound-healing signals. This promotes fibrogenic repair, which attempts to nurture the outgrowth of hepatocyte replacements. However, repair is destined to be futile or maladaptive unless the hepatocyte population is able to activate *Smo* and prevent further lipotoxicity. LSEC, liver sinusoidal endothelial cell.

However, cross-sectional studies in humans cannot establish disease causality. To address this issue, we manipulated *Smo* activity specifically in hepatocytes of healthy adult mice, analyzed transcriptome-wide responses at the single cell level, and performed studies in intact mice and primary mouse hepatocytes to (1) determine the functional significance of Hedgehog pathway dysregulation in NAFLD and (2) identify mechanisms to explain the findings. The present mouse work extends recent studies from our group that showed that conditional deletion of *Smo* specifically in adult hepatocytes rapidly disrupts hepatocyte metabolism of cholesterol and is sufficient to cause hepatic cholesterol accumulation, dyslipidemia, and altered bile acid pool size and composition.^18^ These abnormalities are common in obese patients with the MetS and NAFLD and have been implicated in NASH pathogenesis.^78^^,^^79^ Our latest work further supports the concept that hepatocyte *Smo* depletion potentiates liver lipotoxicity by promoting hepatic accumulation of free fatty acids and increasing oxidative stress, factors that promote progression from hepatic steatosis to steatohepatitis. We identified a plausible mechanism for the former, namely activation of the mTOR-SREB1c signaling axis with consequent induction of SREBP1c lipogenic target genes (Figure 13). A likely mechanism for oxidant stress was also demonstrated, ie, disruption of oxidative phosphorylation secondary to reduced expression of mitochondrial genes that encode electron transport chain components. Importantly, hepatocyte mitochondrial dysfunction inferred from transcriptomic analyses was confirmed by Seahorse studies that demonstrated impaired mitochondrial respiration. This abnormality, in turn, was linked with further functional consequences by immunostaining liver sections to reveal increased oxidative DNA damage in hepatocytes of *Smo*-depleted mice. Oxidative injury to hepatocytes likely contributed to mild aminotransferase elevations that we noted in *Smo*-depleted mice. Another robust MetS-related phenotype of mice with *Smo*-depleted hepatocytes is insulin resistance. GO and GSEA of hepatocyte bulk and single-cell RNA-sequencing data revealed suppression of genes involved in insulin signaling and glucose homeostasis. Insulin challenge experiments identified blunted insulin-mediated phosphorylation of hepatic IRs, increased inhibitory phosphorylation of IRS1/IRS2, inhibited AKT phosphorylation, and reduced phosphorylation and nuclear exclusion of FOXO1, the AKT-regulated transcription factor that promotes transcription of gluconeogenic genes.^27^ Inhibited insulin signaling associated with expected increases in mRNA expression of gluconeogenic genes. Additional insulin challenge experiments established the functional significance of these findings by demonstrating that *Smo* depletion inhibited insulin’s ability to suppress blood glucose. Hyperinsulinemia is the expected compensatory response to reduced insulin activity^80^ and we noted that serum insulin levels were significantly increased in *Smo*-KO mice relative to age- and sex-matched control mice.

Together, our results indicate that hepatocytes can adapt to survive *Smo* inhibition and resultant lipotoxicity but become burdened with dysfunctional mitochondria and oxidative DNA damage. DNA damage triggers mechanisms for cell cycle arrest; thus, new evidence that *Smo*-depletion causes double strand DNA breaks helps to explain other recent work from our group that showed that the regenerative capacity of *Smo*-depleted hepatocytes is significantly reduced.^34^ In turn, the regeneration-inhibited hepatocyte phenotype that results from *Smo* depletion may contribute to NAFLD progression as maladaptive repair is thought to drive progressive liver degeneration that eventuates in cirrhosis. Our comparison of single-cell transcriptomic data from control and *Smo*-depleted hepatocytes support this conjecture. *Smo*-depleted hepatocytes are enriched for genes involved in paracrine processes that regulate tissue repair, and growth-arrested cells that accumulate in injured tissues are known to secrete suites of factors that orchestrate wound healing responses.^81^, ^82^, ^83^

Further research is needed to determine if the pleiotropic effects of *Smo* deletion might derive from a single, primary defect (eg, disruption of cholesterol homeostasis and resultant changes in membrane fluidity that broadly impact signaling and organelle function). Additional studies are also needed to determine if the observed abnormalities in *Smo*-depleted hepatocytes were caused by dysregulating canonical or noncanonical Hedgehog signaling. *Smo* regulates both pathways^84^; studies in nonliver cells indicate that *Smo* can modulate these processes both differentially and independently,^16^ and prior publications by our group and others have reported that inhibiting *Smo* in hepatocytes suppresses activation of AMPK (a key target of noncanonical Hedgehog signaling)^18^ but can also change the *Gli* code (the ultimate effector of canonical Hedgehog signaling).^17^ Finally, the mechanisms responsible for maintaining hepatocyte *Smo* activity in healthy mouse livers and inhibiting *Smo* in the livers of NAFLD patients remain unknown. Matz-Soja et al^17^ have detected expression of Indian hedgehog ligand (Ihh) in perivenous hepatocytes of healthy adult mice and suggested that these hepatocyte-derived ligands may be released into the systemic circulation to function as hepatokines to regulate metabolism and cell state transitions in other organs.^85^ We have been unable to demonstrate Hh ligand mRNAs in healthy adult mouse hepatocytes. However, we did previously report expression of Ihh or Shh by other types of cells that are resident in healthy livers and showed that these factors are readily detected and biologically active in liver-derived microparticles and exosomes.^86^, ^87^, ^88^ Thus, homeostatic *Smo* activity might be regulated by paracrine crosstalk between hepatic sinusoidal cells and hepatocytes, as well as by endocrine-like actions of Hedgehog ligands that associate with liver-derived lipid particles. Hedgehog ligands have already been reported to associate with lipoprotein particles that carry lipid moieties that either activate or inhibit *Smo*, including cholesterol, oxysterols and endocannabinoids.^89^, ^90^, ^91^ Although the source of human lipid particle–associated Hedgehog ligands is uncertain, in *Drosophila* ligand generation occurs in proximal intestinal epithelial cells. These gut-derived ligands associate with lipid particles that are carried into the fat body, where they function as hormones to regulate lipid and glucose metabolism, as well as net adiposity.^92^ A recently published single-cell analysis of the human gastrointestinal tract identified Ihh transcripts in some gut epithelial cells.^93^ Together, these data raise the intriguing possibility that mammals may also rely on the Hedgehog pathway to couple nutrient sensing with coordinated metabolic responses that assure systemic energy balance. Further research to clarify this issue is justified given the importance of the gut-liver axis and the liver-adipose tissue axis in the pathogenesis of NASH and other obesity-related metabolic diseases.

## Materials and Methods

### Mice

Adult male *Smo*^tm2Amc^/J (*Smo*-flox) mice on a C57Bl6/J background (JAX stock# 004526; The Jackson Laboratory, Bar Harbor, ME) (n = 49 mice)^94^ were maintained at Duke University in a temperature-controlled, specific pathogen–free room on 12-hour light–dark cycles with ad libitum access to water and diet. Animal care and surgical procedures were conducted in compliance with an approved Duke University Institutional Animal Care and Use Commitee protocol, and those set forth in the Guide for the Care and Use of Laboratory Animals as published by the National Research Council. At 12 weeks of age, mice were injected by tail vein with 5 × 10^11^ genome equivalents of AAV8-TBG-Luciferase (control [*Smo*^+^]) or AAV8-TBG-Cre Recombinase (*Smo*-KO) to selectively delete the *Smo* gene in hepatocytes.^95^^,^^96^ Viruses were obtained from the University of Pennsylvania Viral Vector Core and Addgene (Watertown, MA). To confirm and extend findings, 7 separate studies were conducted to determine how *Smo* deletion influenced NAFLD pathogenesis. In the first study, *Smo*^+^ (n = 3) and *Smo*-KO (n = 3) mice were fed a Purina 5053 standard chow diet (0.02% w/w; LabDiet, St Louis, MO) for 7 days. In the second study, *Smo*^+^ (n = 4) and *Smo*-KO (n = 3) mice were fed standard chow diet for 10 days. Because responses were similar 7 and 10 days after vector delivery, all subsequent studies were done 7 days after chow-fed mice were treated with the vectors. In the third study, *Smo*^+^ (n = 4) and *Smo*-KO (n = 5) mice were injected with vehicle or insulin after an overnight fast, and livers were harvested 5 minutes later. In the fourth study, *Smo*^+^ (n = 6) and *Smo*-KO (n = 9) mice were fasted for 4 hours before injecting with vehicle or insulin, and serial blood glucose levels were obtained over a 30-minute interval. In the fifth study, primary hepatocytes were isolated from additional *Smo*^+^ (n = 2) and *Smo*-KO (n = 2) mice. In the sixth study, primary hepatocytes were isolated from another 2 *Smo*^+^ and *Smo*-KO mice. In the seventh study, primary hepatocytes isolated from *Smo*^+^ and *Smo*-KO mice (n = 3/group) were subjected to analysis by Seahorse. At whole liver harvest, slices of liver were formalin-fixed for paraffin embedding and the remainder snap-frozen in liquid nitrogen for RNA and protein analysis. Serum was also collected at the time of sacrifice.

### Primary Hepatocyte Isolation

To obtain primary hepatocytes, livers were perfused with collagenase as described.^97^ Hepatocyte preparations were evaluated for viability and purity by light microscopy to assure that viability was at least 95%. Half of the freshly isolated hepatocytes were immediately processed for measuring free fatty acids which were quantified using the Free Fatty Acid Assay Kit (Cat. # ab65341; Abcam, Cambridge, MA), and the remainder were used to obtain RNAs and proteins.

### Immunoblotting and Immunohistochemistry

Liver tissue or isolated hepatocytes from *Smo*^+^ and *Smo*-KO mice were lysed in RIPA buffer (Cat. #R0278; Sigma-Aldrich) supplemented with phosphatase inhibitors and Complete Protease Inhibitor cocktail. Proteins were separated by sodium dodecyl sulfate polyacrylamide gel electrophoresis and transferred electrophoretically to nitrocellulose membranes (Cat. #1620112; Bio-Rad, Hercules, CA). Blots were blocked, incubated OVN at 4°C with primary antibodies, probed with secondary horseradish peroxidase–conjugated antibodies, and visualized by enhanced chemiliuminesence with detection on a Chemidoc MP Imaging system (Cat. #17001402; Bio-Rad).

Formalin-fixed, paraffin-embedded liver tissue was cut into 5-μm serial sections and placed on glass slides. For immunohistochemistry, sections were deparaffinized with xylene, rehydrated with ethanol, and incubated for 10 minutes in 3% hydrogen peroxide to block endogenous peroxidase. Antigen retrieval was performed by heating in 10 mM sodium citrate (pH 6.0). Sections were blocked in Dako protein block solution (Cat. #X9090; Agilent) for 1 hr and incubated OVN at 4°C with indicated primary antibodies. Horseradish peroxidase–conjugated anti-rabbit (Cat. #K400311-2; Agilent) and anti-mouse (Cat. #K400111-2; Agilent) secondary antibodies were used to visualize target proteins. DAB reagent (Cat. #K346811-2; Agilent) was applied in the detection procedure. Tissue sections were counterstained with Aqua Hematoxylin-INNOVEX (Innovex Biosciences, Richmond, CA). Omitting primary antibodies from reactions eliminated staining, demonstrating specificity. For phospho-FOXO1 Serine 256 staining, the numbers of cells with stained cytoplasms were counted in 10 randomly chosen ×20 fields per section per mouse. Immunohistochemistry antibodies and reverse-transcription polymerase chain reaction (primer sequences used in this study are specified in Tables 3 and 4.

**Table 3:** List of Antibodies for Western Blotting and IHC

Primary Antibody	Supplier	Reference	Dilution
β-tubulin	Abcam	Cat. #ab6046	1:3000
SREBP2	Abcam	Cat. #ab30682	1:1000
pAkt Ser 473	Cell Signaling	Cat. #4060	1:1000
Total Akt	Cell Signaling	Cat. #9272	1:1000
pFOXO1 Ser 256	Sigma Aldrich	Cat. #SAB4300094-100UG	1:1000
Total FOXO1	Cell Signaling	Cat. #2880	1:1000
pIRS1 Ser 307	Thermo Fisher Scientific	Cat. #PA1-1054	1:1000
Total IRS1	Upstate	Cat. #06-248	1:1000
pIRS2 Ser 731	GeneTex	Cat. #GTX23690	1:1000
Total IRS2	Upstate	Cat. #06-506	1:1000
pIRβ Tyr 1150/1151	Cell Signaling Technology	Cat. #3024S	1:1000
Total insulin receptor β	Cell Signaling Technology	Cat. #3025S	1:1000
pmTOR Ser 2448	Cell Signaling Technology	Cat. #2971	1:1000
Total mTOR	Cell Signaling Technology	Cat. #2972S	1:1000
pP70S6K Thr 389	Cell Signaling Technology	Cat. #9205S	1:3000
Total P70S6K	Cell Signaling Technology	Cat. #9202	1:1000
FGF15	Santa Cruz Biotechnology	Cat. #sc-514647	1:1000
pFOXO1 Ser 256 (IHC)	Sigma-Aldrich	Cat. #SAB4300094-100UG	1:200

IHC, immunohistochemistry.

**Table 4:** *Mus musculus* Primer Sequences Used

Gene	Forward Primer 5′ to 3′	Reverse Primer 5′ to 3′
*FOXO1*	ACATTTCGTCCTCGAACCAGCTCA	ATTTCAGACAGACTGGGCAGCGTA
*G6PC*	GTGCAGCTGAACGTCTGTCTGTC	TCCGGAGGCTGGCATTGTA
*HES1*	AGAGGCTGCCAAGGTTTTTG	TCCCACTGTTGCTGGTGTAGA
*HEY1*	CCGAAGTTGCCCGTTAT	CGCTGGGATGCGTAGTT
*IGFBP1*	AGATCGCCGACCTCAAGAAA	CCAGGGATGTCTCACACTGT
*NOTCH1*	TGGCCTCAATGGGTACAAGT	AAGGGTTGGACTCACACTCG
*NOTCH2*	ACGGTCTTGACTTCTGCG	ATGGTGCTCTTGGTGTTGG
*NOTCH4*	GTGAGAAAGAAGTGGACGAATG	GAAACCAGGACGGCAGAG
*PCK1*	CCACAGCTGCTGCAGAACAC	GAAGGGTCGCATGGCAAA
*Smo*	GCCTGGTGCTTATTGTGG	GGTGGTTGCTCTTGATGG

### Serum Analysis

Serum insulin was measured with an Ultra-Sensitive Mouse Insulin ELISA kit (Cat. #90080; Crystal Chem, Elk Grove Village, IL). Serum alanine aminotransferase and serum aspartate aminotransferase were measured using alanine aminotransferase (SGPT) and AST (SGOT) kits from Biotron Diagnostics (Hemet, CA).

### Insulin Sensitivity Assay

For analysis of acute insulin sensitivity, mice were fasted for 14 hours. General anesthesia was induced using isoflurane, and the abdomen was opened through a midline incision and the portal vein identified. Insulin (1 μmol/L; Cat. #12585014; Thermo Fisher Scientific, Waltham, MA) or carrier (0.9% NaCl and 0.1% bovine serum albumin) were injected into the portal vein using a 28-gauge needle; mice were killed 5 minutes following portal vein injection, and livers were dissected and placed in liquid nitrogen within 15 seconds.

### Insulin Tolerance Test

Insulin tolerance test was performed as previously described.^98^ Mice were fasted for 4 hours before insulin injection (0.75 IU insulin/kg body weight). Blood glucose was measured at 0, 15, and 30 minutes after insulin injection using an Accu-Chek glucometer (Accu-Chek, Corydon, IN).

### Mitochondrial Respiration Assay

Primary hepatocytes were isolated and mitochondrial bioenergetics were assessed in primary hepatocytes using a Seahorse XCFa analyzer (Agilent) as described.^34^ Every sample was analyzed in triplicate. The Seahorse XF Wave software was used to group the respiration data from separate mice into a single representative curve and data was normalized to mitochondrial protein content.

### Human Liver Samples

We utilized frozen liver biopsies prospectively collected from human subjects (n = 368) with no histologic features of chronic liver disease (n = 69) or biopsy-proven NAFLD (n = 299) and archived in the Duke University Health System (DUHS) NAFLD Clinical Database and Biorepository. A summary of patient characteristics is detailed in Table 2. The DUHS NAFLD Clinical Database and Biorepository is approved by our Institutional Review Board (Pro00005368). The DUHS NAFLD Clinical Database and Biorepository contains clinical data and biospecimens from NAFLD patients who underwent a diagnostic liver biopsy to grade and stage the severity of presumed NAFLD as part of standard of care. Specimens were collected at the time of standard of care liver biopsy following a 12-hour fast. Systematic chart review and data extraction was performed. Only patients who consented to utilize their samples for “-omics” analysis were included in our analysis. The primary outcome for the analysis was fibrosis stage. All liver biopsy specimens were stained with hematoxylin and eosin and Masson’s trichrome and graded and staged by an experienced hepatic pathologist (C.G.) according to the published NASH Clinical Research Network scoring system.^99^ For the analyses, fibrosis stages 1a, 1b, and 1c were combined and treated as stage 1 fibrosis. For the present study, NAFLD was defined as (1) presence of >5% hepatic steatosis on liver biopsy, (2) absence of histologic and serologic evidence for other forms of chronic liver disease (chronic viral hepatitis, autoimmune liver disease, alpha-1-antitrypsin deficiency, genetic hemochromatosis, Wilson's disease), and (3) little or no alcohol consumption (<20 g/d for women and <30 g/d for men). Clinical data were obtained at the time of liver biopsy and included height, weight, body mass index (BMI), age, sex, race, ethnicity, the presence of hypertension, diabetes mellitus, hyperlipidemia, and dyslipidemia. Obesity was diagnosed when BMI was ≥30 kg/m^2^, and overweight when BMI was ≥25 and <30 kg/m^2^. The presence of hypertension was defined by known diagnosis in the medical record and/or the use of any antihypertensive medication. The presence of diabetes mellitus was defined by known diagnosis in the medical record, use of any insulin-sensitizing agent or insulin, and/or a glycosylated hemoglobin value of >6.5%. Hyperlipidemia was defined as a total cholesterol level >200 mg/dL, an low-density lipoprotein cholesterol >120 mg/dL or use of a lipid-lowering agent.

### Processing and Analysis of Single-Cell RNA-Seq Data

Freshly isolated hepatocytes were washed and re-suspended in 0.04% UltraPure bovine serum albumin and counted using the automated cell counter. GEM generation, post GEM-RT cleanup, complementary DNA amplification, and library construction were performed following 10x Genomics Single Cell 3′ v2 chemistry (10x Genomics, Pleasanton, CA). Libraries were sequenced on Illumina NovaSeq PE150 (Illumina, San Diego, CA). A total of 500 million paired-end reads were obtained for each sample as recommended. Cell Ranger from 10x Genomics was used for initial data processing. Cell-by-gene expression matrices were generated with valid cell barcodes. Low quality cells with high mitochondrial contents (≥ 60%) and possible doublets were excluded. A total of ∼9500 cells were retained for further analysis with a median of 2368 genes detected per cell. Prior to combining control and knockout libraries, batch correction was performed using RISC software (Hagenburg im Mühlkreis, Austria) with default parameters.^100^ The integrated dataset was then converted to a Seurat object and visualized in UMAP with a 0.5 resolution.^101^ Cluster identities were annotated using cell type–specific markers for hepatocytes, Kupffer cells, and endothelial cells from Halpern et al*.*^30^ Pathways enriched in specific clusters were analyzed using the GSEA software from the Broad Institute (Boston, MA).^102^

### Bulk RNA-Seq and Analysis

Total RNAs were isolated from liver tissues or hepatocytes as previously described.^103^ Libraries were made with NEBNext Ultra RNA Library Prep Kit from Illumina. Sample qualities were assessed by Agilent RNA Bioanalyzer chip traces before pooled and sequenced with an Illumina HiSeq 2500. Sequencing reads were trimmed using TrimGalore (http://www.bioinformatics.babraham.ac.uk/projects/trim_galore/) and aligned to the reference genome using STAR.^104^ Gene expression counts were generated using HTSeq^105^ and DESeq2^106^ was used to perform differential expression analyses (absolute log2 fold change ≥ 1 and *q* <0.05). Receiver-operating characteristic was used for assessing the diagnostic ability of *Smo* expression in discriminating F3/F4 vs healthy, or F3/F4 vs F0/F1. About one-quarter of cases (∼55–65 samples) were reserved for training a logistic regression model, and the remaining three-quarters of cases were for test set. Receiver-operating characteristic curves and scores were generated using pROC package in R.^107^

Nonhepatocyte cell-type libraries from multiple sources were compiled. Libraries received only linear normalization so that the proportions of individual transcripts within the total library were unchanged. Normalizations to the total transcripts in the library (the total cell-specific transcriptome) and to the median transcript value of each cell library were both useful for comparisons. FASTQ datasets were aligned to the mm9 genome. Raw reads were quantified using the RefSeq annotation and GenPlay software.^108^ Extraneous transcripts (eg, ribosomal RNA, mitochondrial RNA, and small RNA) were removed, and genes with multiple transcripts were simplified to the most abundant isoform. Quantifications were averaged for cell types with multiple libraries. Read counts were then converted to molecule counts (reads/kb), by dividing by transcript length (kb). Those with good sequence depth and uniform full-length transcripts received further analysis. Non–liver cell libraries were compared for linear correlation with whole liver libraries. Among numerous published libraries of fibroblasts and macrophage types, tail fibroblast^109^ and peritoneal macrophage^110^ libraries were included because they showed the strongest correlations with whole liver. Libraries from multiple B cell and T cell subtypes showed good correlations with liver, but there was insufficient information to distinguish the subtypes in normal liver. The selected libraries were from quiescent splenic follicular B cells^111^ and unstimulated peripheral blood CTL.^112^

### Cell-Specific Expression Within the Liver Transcriptome

To assess the gene expression of individual cell types, deep RNA-seq libraries from liver (whole liver, cholangiocytes, sinusoidal endothelium, stellate cells, and Kupffer cells) and from other tissues (fibroblasts, macrophages, monocytes, neutrophils, B cells, and T cells) were aligned, quantified, filtered, and normalized. Sets of genes specific for each cell type were used to calculate the proportion of that cell type within whole liver. These proportions were then used to calculate the contribution of each cell type to the total expression of genes expressed in multiple cell types.

### Cell Counting Profiling

Comparison of 10 cell-type libraries with each other and with whole liver libraries identified sets of genes uniquely expressed in single cell types. Within a single library, each transcript (t) is a fraction of the total transcriptome (T). If the transcript is unique to one cell type, the ratio of its fraction in the total liver transcriptome (t_L_/T_L_) to its fraction in the cell-type transcriptome (t_C_/T_C_) represents the fraction of the cell-specific transcriptome within the total liver transcriptome. All 4 of these values are readily measured, and if libraries are normalized by total transcripts, then T_L_ and T_C_ cancel. However, note that the cell transcriptome fraction of liver is not the same as the cell number fraction, as different cell types do not have the same number of transcripts. The set of ratios for each cell type was averaged to approximate the proportion of its RNA within the whole liver transcriptome. The values of these cell-type fractions were summed to calculate the nonparenchymal cell (NPC) fraction, and then the hepatocyte fraction was calculated by subtracting the NPC value from the whole liver value. Although isolated hepatocyte transcriptomes were compared, they could not be used for compilation for 2 reasons: (1) many reactive genes were strongly induced by hepatocyte isolation and (2) isolated hepatocytes retained a substantial fraction of NPC.

### Data Availability

All primary sequencing data have been deposited in the Gene Expression Omnibus under accession number GSE213623 (bulk RNA-seq) and GSE213183 (single-cell RNA-seq). For cell type–specific gene expression analysis in nonparenchymal cells, the following mouse RNA-seq libraries were used and downloaded from the Sequence Read Archive National Center for Biotechnology Information database: whole liver (SRR6335223, SRR6335224) and isolated hepatocytes (SRS2725640, SRS3489136)^113^; cholangiocyte (SRR6392083, SRR6392084, SRR6392085)^114^; sinusoidal endothelium (SRR5920415, SRR5920416, SRR5920417)^115^; fibroblast (SRR847341)^109^; Kupffer cell (SRR7519573, SRR7519573, SRR7519573)^116^; peritoneal macrophage (SRR1177046, SRR1177047)^110^; monocyte (SRR7163783, SRR7163784, SRR7163785)^117^; neutrophil (SRR1177062, SRR1177063)^110^; quiescent follicular B cells (SRR3724513, SRR3724514)^111^; and unstimulated cytotoxic T lymphocytes (SRR5520192).^112^

### Statistical Analysis

Statistical analysis was performed by GraphPad Prism 5 software (GraphPad Software, San Diego, CA). All data were expressed as mean ± SEM. Statistical significance of differences was determined between 2 groups using unpaired Student’s *t* test and 2-way repeated measures analysis of variance. Differences with *P* ≤ .05 were considered statistically significant. Fisher’s exact test was applied, and *P* values were indicated in the corresponding figures. Pearson correlation was used to measure the strength of relationship between 2 independent datasets.
